# Unique roles of the unfolded protein response pathway in fungal development and differentiation

**DOI:** 10.1038/srep33413

**Published:** 2016-09-15

**Authors:** Kwang-Woo Jung, Yee-Seul So, Yong-Sun Bahn

**Affiliations:** 1 Department of Biotechnology, Yonsei University, Seoul 03722, Republic of Korea

## Abstract

*Cryptococcus neoformans*, a global fungal meningitis pathogen, employs the unfolded protein response pathway. This pathway, which consists of an evolutionarily conserved Ire1 kinase/endoribonuclease and a unique transcription factor (Hxl1), modulates the endoplasmic reticulum stress response and pathogenicity. Here, we report that the unfolded protein response pathway governs sexual and unisexual differentiation of *C. neoformans* in an Ire1-dependent but Hxl1-independent manner. The *ire1*∆ mutants showed defects in sexual mating, with reduced cell fusion and pheromone-mediated formation of the conjugation tube. Unexpectedly, these mating defects did not result from defective pheromone production because expression of the mating pheromone gene (*MFα1*) was strongly induced in the *ire1*∆ mutant. Ire1 controls sexual differentiation by modulating the function of the molecular chaperone Kar2 and by regulating mating-induced localisation of mating pheromone transporter (Ste6) and receptor (Ste3/Cprα). Deletion of *IRE1*, but not *HXL1*, also caused significant defects in unisexual differentiation in a Kar2-independent manner. Moreover, we showed that Rim101 is a novel downstream factor of Ire1 for production of the capsule, which is a unique structural determinant of *C. neoformans* virulence. Therefore, Ire1 uniquely regulates fungal development and differentiation in an Hxl1-independent manner.

The endoplasmic reticulum (ER) in eukaryotic cells is a central organelle where most secreted and transmembrane proteins undergo proper folding and post-translational modification. A variety of conditions, including alteration of calcium homeostasis, nutrition starvation, and pathogen infection, lead to the accumulation of unfolded or misfolded proteins, which causes ER stress. When proteins accumulate, cells activate the evolutionarily conserved unfolded protein response (UPR) to mitigate the ER stress by up-regulating the expression of a diverse set of genes encoding ER chaperones and folding enzymes^1^,^2^. In the model yeast *Saccharomyces cerevisiae*, the UPR pathway consists of Ire1, which is a Ser/Thr kinase with an endonuclease domain, and its downstream transcription factor (TF) Hac1. When *S. cerevisiae* cells encounter ER stress, activated Ire1 endonuclease removes an unconventional intron from *HAC1* mRNA^3^. This unconventionally spliced form of *HAC1* mRNA is translated into an active bZIP TF. Activated Hac1 is translocated into the nucleus where it induces the expression of UPR target genes^4^.

The roles of the UPR pathway in the cellular responses against diverse environmental stressors in fungi have been well characterised. However, the cellular functions of the UPR pathway in differentiation and morphological development is poorly understood. In *S. cerevisiae*, during nitrogen starvation and depending on the fermentable carbon source, morphological changes are triggered in diploid cells, inducing a pseudohyphal form^5^. Activation of the UPR represses both pseudohyphal growth and sporulation by regulating early meiotic genes^5^. In the corn smut fungus *Ustilago maydis*, deletion of *IRE1* results in reduced filamentous growth, whereas perturbation of *CIB1*, which is a *HAC1* homolog, does not affect filamentous growth^6^. In the human fungal pathogen *Candida albicans*, hyphal development is decreased in a *hac1*∆ mutant during serum stimulation. However, the function of Ire1 in hyphal development has not been characterised^7^. In the rice blast fungus *Magnaporthe oryzae*, Hac1 is essential for conidia production in asexual development; however, the regulatory mechanism involving the UPR pathway during asexual development has not been fully characterised^8^.

*Cryptococcus neoformans*, which belongs to the phylum Basidiomycota and causes fatal meningoencephalitis in humans, has a haploid genome, except for interspecies (*C. neoformans* x *Cryptococcus gattii*)^9^ and interserotype (AD)^10^ hybrids, with a bipolar mating-type system consisting of α and **a** mating types. Two types of sexual differentiation exist in *C. neoformans*; one is a sexual (heterothallic or opposite sex) mating system, and the other is a unisexual (homothallic or same sex) mating system^11^. In the opposite sex mating system, cells of the opposite mating type are recognized via their secreted pheromone. Then, the cells strongly produce a mating pheromone, which triggers cell-cell fusion (plasmogamy)^11^. In filaments with fused clamp connections, the two nuclei in the cells of opposite mating types migrate as an alternating pair and reach the basidium, where nuclear fusion (karyogamy) occurs. Then, the diploid nucleus undergoes meiosis to produce four haploid nuclei and basidiospores^11^. Intriguingly, cells of the α mating type are predominant in natural and clinical environments^12^, suggesting that sexual mating may not be a frequent event in nature. *C. neoformans* undergoes monokaryotic fruiting from identical mating type (*MAT*α or *MAT***a**) cells^13^,^14^,^15^. Recent studies reported that the production of monokaryotic hyphae is induced either asexually or by same-sex mating^16^,^17^. During same-sex mating, recombination occurs at levels similar to that in sexual mating, and ploidy change occurs. Deletion of *DMC1*, which encodes a meiosis-specific recombinase, impairs sporulation during monokaryotic fruiting^16^. Monokaryotic fruiting can also occur in a same-sex mating-independent manner, when cells are arrested in the G2 stage of the cell cycle by high temperature^17^.

Recently, we reported that the *C. neoformans* UPR pathway consists of an evolutionarily conserved Ire1 kinase/endonuclease and a unique bZIP TF, Hxl1, which is structurally and phylogenetically distinct from yeast Hac1/human XBP1^18^. We also demonstrated that the *Cryptococcus* UPR pathway is required for the ER stress response, thermotolerance, cell wall integrity, antifungal drug resistance, and pathogenicity^18^. Subsequently, we identified and functionally characterised an ER-resident molecular chaperone (Kar2/BiP) as one of the downstream targets of the *Cryptococcus* UPR pathway^19^. Nevertheless, the roles of the UPR pathway in *Cryptococcus* differentiation remain uncharacterised. Here, we found that Ire1 controls both sexual and unisexual differentiation in an Hxl1-independent manner. Ire1 modulates sexual differentiation by enhancing molecular chaperone function and by controlling the transcellular localisation of mating pheromone transporter (Ste6) and receptor (Ste3; also known as Cprα). Furthermore, we found that the UPR pathway utilises Rim101 to control production of the polysaccharide capsule, which is a critical cell surface structure and a key virulence determinant. Therefore, the current study points to a novel role for the UPR pathway in fungal differentiation and development.

## Results

### Ire1 kinase, but not the Hxl1 transcription factor, in the UPR pathway is involved in the sexual differentiation of *C. neoformans*

**Figure 1:**
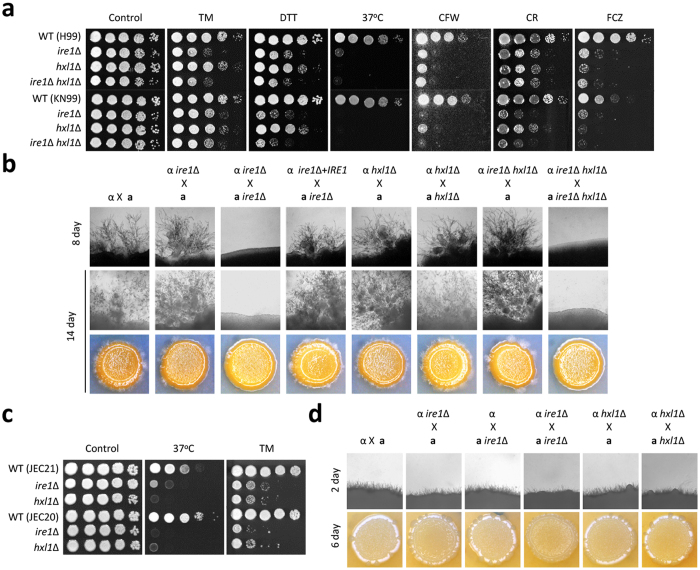
The UPR pathways in *Cryptococcus* serotypes A and D play conserved roles in thermotolerance, ER stress, and sexual differentiation. (**a**) *Cryptococcus* serotype A strains were cultured in liquid YPD medium at 30 °C overnight. Cells were serially diluted 10-fold (1 to 10^4^), and then spotted onto YPD medium containing stress-inducing agents [Calcofluor white (CFW) 5 mg/mL, Congo red (CR) 1%, tunicamycin (TM) 0.03 μg/mL, and dithiothreitol (DTT) 1 mM] or antifungal drugs [fluconazole (FCZ) 10 μg/mL]. For the temperature sensitivity assay, 10-fold serially diluted cells were spotted onto YPD medium and then incubated at 37 °C for 2 d–4 d. (**b**) Serotype A strains were co-cultured on V8 medium (pH 5.0) for 2 weeks at room temperature in the dark: α (H99) × **a** (KN99**a**), α *ire1*∆ (YSB552) × **a** (KN99**a**), α *ire1*∆ (YSB552) × **a** *ire1*∆ (YSB550), α *ire1*∆ + *IRE1* (YSB1000) × **a** *ire1*∆ (YSB550), α *hxl1*∆ (YSB723) × **a** (KN99**a**), α *hxl1*∆ (YSB723) × **a** *hxl1*∆ (YSB850), α *ire1*∆ *hxl1*∆ (KW308) × **a** (KN99**a**), and α *ire1*∆ *hxl1*∆ (KW308) × **a** *ire1*∆ *hxl1*∆ (KW350). (**c**) *Cryptococcus* serotype D strains were grown in liquid YPD medium at 30 °C for 16 h. *Cryptococcus* strains were serially diluted 10-fold and spotted on YPD medium containing an ER stress inducer (TM 0.2 μg/mL). The cells were incubated at 30 °C for 2 d–4 d and photographed daily. For the thermotolerance assay, serially diluted cells were spotted onto YPD medium and then incubated at 37 °C for 2 d–4 d. (**d**) Serotype D *MAT*α and *MAT***a** strains were co-cultured on V8 medium (pH 7) for 6 d at room temperature in the dark: α (JEC21) × **a** (JEC20), α *ire1*∆ (YSB2886) × **a** (JEC20), α (JEC21) × **a** *ire1*∆ (YSB2026), α *ire1*∆ (YSB2886) × **a** *ire1*∆ (YSB2026), α *hxl1*∆ (YSB2030) × **a** (JEC20), and α *hxl1*∆ (YSB2030) × **a** *hxl1*∆ (YSB1985). Representative edges of the mating patches were photographed at 100× magnification.

To test whether the UPR is involved in the sexual differentiation of *C. neoformans*, we first constructed *MAT***a** *ire1*∆ and *hxl1*∆ mutants in a serotype A KN99**a** (wild-type *MAT***a**) genetic background, for comparison with the *ire1*∆ and *hxl1*∆ mutants that were previously constructed in a serotype A H99 (wild-type *MAT*α) strain background^18^. The *MAT***a** *ire1*∆ and *hxl1*∆ mutants showed severe growth defects at 37 °C and were susceptible to cell wall-destabilizing agents (calcofluor white and Congo red), ER stress inducers (tunicamycin and dithiothreitol), and azole drugs (Fig. 1a). These phenomena are similar to the reported phenotypes of *MAT*α *ire1*∆ and *hxl1*∆ mutants^18^, suggesting that the conventional roles of the UPR components are highly conserved in cells of the opposite mating types in serotype A *C. neoformans*.

The *MAT*α *ire1*Δ and *hxl1*Δ mutants showed wild-type levels of filamentation in unilateral crosses with the KN99**a** strain (Fig. 1b). However, in a bilateral cross (α *ire1*Δ × **a** *ire1*Δ), the *ire1*Δ mutants showed severe defects in filament formation, whereas the *hxl1*Δ mutants did not show any filamentation defects (Fig. 1b). This phenomenon is in stark contrast to the finding that Ire1 and Hxl1 mutants share a variety of phenotypes, as mentioned above. Corroborating this finding, the filamentation defect observed in the bilateral cross with the α *ire1*∆ and **a** *ire1*∆ mutants was restored to wild-type levels by introducing the wild-type *IRE1* gene into the α *ire1*∆ mutant (Fig. 1b). To further demonstrate that Hxl1 is dispensable for filamentation, we generated *ire1* Δ *hxl1*Δ double mutants. In terms of growth defects in response to ER stress, cell wall stress, azole drug treatment, and high temperature (37 °C), the *ire1*∆ *hxl1*∆ double mutant was similar to the *ire1*∆ mutant (Fig. 1a). Deletion of *HXL1* did not further exacerbate the filamentation defects in the *ire1*Δ mutant, as shown by the finding that the *ire1*Δ and *ire1*Δ *hxl1*Δ mutants exhibited similar levels of filamentation in a unilateral cross with the wild-type *MAT***a** strain. These data indicate that Ire1 has an Hxl1-independent role in sexual differentiation.

To determine whether the Ire1-dependent, Hxl1-independent role of the UPR pathway in sexual differentiation is a serotype-specific event, we constructed UPR mutants in a serotype D background and tested whether Ire1 also affects sexual mating in serotype D strains. As was observed in the serotype A UPR mutants, both the serotype D *MAT*α and *MAT***a** *ire1*Δ and *hxl1*Δ mutants showed severe growth defects at high temperature (37 °C) and increased susceptibility to ER stressors (Fig. 1c), suggesting that the general UPR function is highly conserved in the two *C. neoformans* varieties. In general, serotype D *MAT*α JEC21 and *MAT***a** JEC20 undergo more robust mating than serotype A H99 and KN99**a** strains. In agreement with the result from the serotype A strain background, deletion of *IRE1* in the serotype D strain reduced sexual mating efficiency during a bilateral cross, whereas deletion of *HXL1* did not affect the mating (Fig. 1d).

In *S. cerevisiae* and *U. maydis*, constitutive activation of the UPR through expression of spliced *HAC1* mRNA represses filamentous growth^5^,^6^. Here, we determined whether constitutive activation of the UPR pathway affects the sexual differentiation of *C. neoformans* using strains harbouring a spliced version of *HXL1*. In contrast to *S. cerevisiae* and *U. maydis*, constitutive activation of the UPR pathway in *C. neoformans* through expression of spliced *HXL1* did not affect sexual differentiation (Supplementary Figure S1).

In conclusion, Ire1 in the UPR pathway regulates sexual differentiation in an Hxl1-independent manner in serotype A and D *C. neoformans*.

### Ire1 is required for cell-cell fusion and construction of the conjugation tube during mating

**Figure 2:**
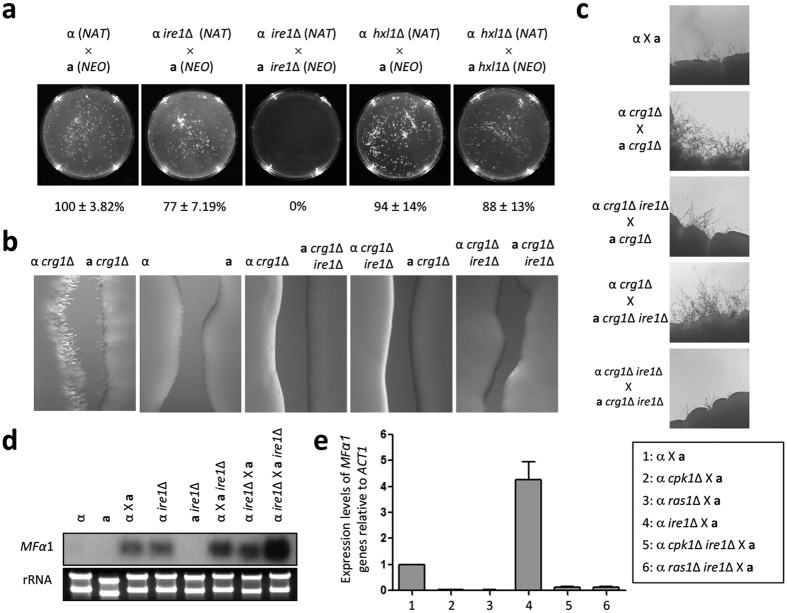
Ire1 positively regulates cell-cell fusion and conjugation tube formation and negatively controls the gene expression of mating pheromone. (**a**) A cell fusion assay was performed with the following strains: α (YSB119) × **a** (YSB121), α *ire1*∆ (YSB552) × **a** (YSB121), α *ire1*∆ (YSB552) × **a** *ire1*∆ (YSB550), α *hxl1*∆ (YSB723) × **a** (YSB121), and α *hxl1*∆ (YSB723) × **a** *hxl1*∆ (YSB850). The cell fusion percentage on each plate relative to that for the α (YSB119) × **a** (YSB121) cross was calculated. (**b,c**) Confrontation and mating assays were performed with the following strains: α (H99), **a** (KN99**a**), α *crg1*∆ (H99 crg1), **a** *crg1*∆ (PPW 196), α *crg1*∆ *ire1*∆ (YSB1008), and **a** *crg1*∆ *ire1*∆ (YSB1010). Representative edges of the mating patches were photographed at 100× magnification. (**d,e**) Northern blot or quantitative RT-PCR analysis was employed to monitor the expression levels of the pheromone gene using total RNA isolated from solo cultures or co-cultures of the indicated strain(s) grown for 24 h under mating conditions. Error bars indicate the standard deviation. The strains used for the northern blot and quantitative RT-PCR analyses were as follows: α (H99), **a** (KN99**a**), α *ire1*∆ (YSB552), a *ire1*∆ (YSB550), α *ras1*∆ (YSB53), α *cpk1*∆ (YSB127), α *ras1*∆ *ire1*∆ (YSB2507), and α *cpk1*∆ *ire1*∆ (YSB2505).

The finding that mutation of *IRE1* inhibited filamentation in bilateral crosses of both serotype A and D strains led us to investigate which mating step is regulated by Ire1 kinase. Firstly, we monitored the ability of *ire1*Δ mutants to undergo cell-cell fusion. Quantitative measurement of dikaryotic cell fusion products from unilateral and bilateral crosses with *ire1*Δ mutants showed that the cell fusion efficiency was lower than that of a control cross (Fig. 2a). The cell fusion defect in the bilateral cross between the *ire1*Δ mutants was much more severe than the defect in the unilateral cross. As expected, the *hxl1*Δ mutants exhibited wild-type levels of cell-cell fusion efficiency (Fig. 2a), further supporting the notion that Hxl1 is dispensable for sexual mating.

Next, we assessed conjugation tube formation during the mating response using confrontation assays with pheromone-hypersensitive *crg1*Δ mutants, which are devoid of the RGS protein that desensitises the pheromone response pathway and robustly form conjugation tubes^20^. For this purpose, we deleted the *IRE1* gene in *MAT*α and *MAT***a** *crg1*Δ mutants. When the *MAT*α and *MAT***a** *crg1*Δ *ire1*Δ double mutants were confronted by *MAT***a** and *MAT*α *crg1*Δ mutants, respectively, conjugation tube formation was abrogated (Fig. 2b). Further supporting this finding, deletion of *IRE1* also significantly reduced enhanced filament production between *MAT*α and *MAT***a** *crg1*Δ mutants (Fig. 2c). Thus, Ire1, but not Hxl1, in the UPR pathway positively regulates cell-cell fusion and conjugation tube formation during sexual differentiation of *C. neoformans*.

### The Ire1 kinase represses the expression of the mating pheromone gene in a Cpk1- and Ras1-dependent manner

The finding that Ire1 is required for cell fusion with opposite mating types and conjugation tube production prompted us to test whether Ire1 influences mating pheromone expression at the initial mating step. In contrast to the positive role of Ire1 in cell fusion and conjugation tube formation, deletion of *IRE1* resulted in increased pheromone gene expression in the presence or absence of the opposite mating type (Fig. 2d). In fact, the mating pheromone gene was expressed even when the α *ire1*Δ mutant was cultured alone (Fig. 2d), indicating that Ire1 may repress pheromone gene expression under basal conditions.

The Cpk1/Fus3 mitogen activated protein kinase (MAPK) pathways for pheromone production are evolutionarily well conserved across fungal species^21^,^22^. In addition to the Cpk1/Fus3 MAPK pathways, Ras signalling also regulates the mating pheromone-responsive pathway and differentiation in the fission yeast *Schizosaccharomyces pombe* and the human fungal pathogen *C. albicans*^23^,^24^,^25^. Furthermore, the Cpk1 MAPK and Ras signalling pathways regulate the mating response by controlling the expression of the pheromone gene in *C. neoformans*^21^,^26^. To assess the crosstalk among Ire1 and the Cpk1 and Ras1 pathways, we deleted the *CPK1* and *RAS1* genes in the *ire1*Δ mutant and monitored mating pheromone expression in the *cpk1*Δ *ire1*Δ and *ras1*Δ *ire1*Δ double mutants. Notably, the increased levels of mating pheromone gene expression detected in the *ire1*Δ mutant were significantly reduced in the *cpk1*Δ *ire1*Δ and *ras1*Δ *ire1*Δ double mutants (Fig. 2e), indicating that Ire1 represses the mating response in a Cpk1- and Ras-dependent manner.

### Deletion of *IRE1* resulted in delayed cellular translocation of Ste6 and Ste3, but not Cpr2, from the ER to the cell membrane during mating

The puzzling observation that Ire1 positively regulates cell fusion, conjugation tube formation, and filamentation but negatively regulates pheromone gene expression led us to hypothesise that Ire1 may be involved in the proper localisation of mating-related cell membrane proteins during mating, because most secreted and plasma membrane proteins are synthesised, folded, and matured in the ER. This finding was supported by the observation that in *S. cerevisiae*, the Ste2 mating pheromone receptor and Ste6 pheromone transporter require ER quality control for efficient delivery to the plasma membrane^27^,^28^. During the mating response in *C. neoformans*, the mating pheromone is secreted through Ste6, a mating pheromone transporter, and is recognised by a mating pheromone receptor (Ste3 or Cpr2) in opposite mating type cells^29^,^30^,^31^. We hypothesised that perturbation of ER homeostasis through deletion of *IRE1* leads to changes in the localisation of the mating pheromone transporter or receptors during sexual differentiation.

**Figure 3:**
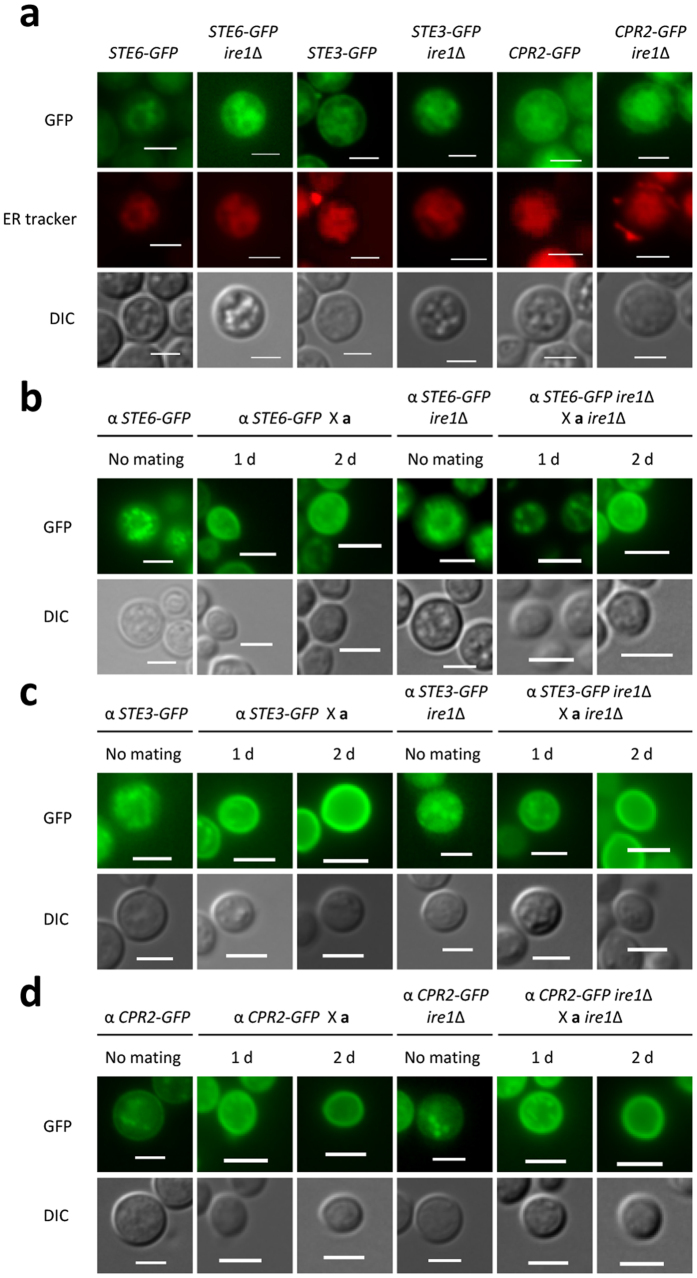
Deletion of *IRE1* resulted in delayed transcellular localisation of Ste6 and Ste3 but not Cpr2. (**a**) Strains harbouring *STE6-GFP*, *STE3-GFP*, or *CPR2-GFP* were cultured in liquid YPD medium at 30 °C overnight, and then fixed with formaldehyde. After fixation, the cells were stained with an ER tracker (E34250; Invitrogen) to visualise the ER. (**b–d**) Strains were cultured in liquid YPD medium at 30 °C overnight. Then, cells harbouring *STE6-GFP*, *STE3-GFP*, or *CPR2-GFP* were co-cultured with KN99**a** or **a** *ire1*∆ (YSB550) cells on V8 medium (pH 5.0) for the indicated time period in the dark. The cellular localisation of each GFP fusion protein was visualised by fluorescence microscopy. The strains used for the mating assay were as follows: **a** (KN99**a**), **a** *ire1*∆ (YSB550), *STE6-GFP* (YSB2619), *STE6-GFP ire1*∆ (YSB2744), *STE3-GFP* (YSB2864), *STE3-GFP ire1*∆ (YSB3020), *CPR2-GFP* (YSB3000), and *CPR2-GFP ire1*∆ (YSB3311).

To test this hypothesis, we first constructed cells expressing *STE6*-*GFP*, *STE3*-*GFP,* or *CPR2*-*GFP* and then monitored the localisation of Ste6-Gfp, Ste3-Gfp, and Cpr2-Gfp during mating. We confirmed that strains harbouring Ste6-Gfp, Ste3-Gfp, and Cpr2-Gfp exhibited wild-type mating efficiency with opposite mating type strains (data not shown). Under basal conditions without mating partners, Ste6, Ste3, and Cpr2 showed mainly punctate localisation in the ER (Fig. 3a). However, after an encounter with the opposite mating partner (KN99**a**), Ste6-Gfp, Ste3-Gfp, and Cpr2-Gfp were translocated to the cell membrane (Fig. 3b–d). This observation is consistent with the localisation of Cpr2 during sexual mating of *C. neoformans*^30^. Here, we show, for the first time, that the cellular localisation of the pheromone transporter and receptors switches from the ER to the cell membrane during sexual differentiation in *C. neoformans*.

Next, to determine whether Ire1 affects the cellular localisation of Ste6, Ste3, and Cpr2, we deleted the *IRE1* gene in cells expressing Ste6-Gfp, Ste3-Gfp, or Cpr2-Gfp. Under basal conditions without mating partners, deletion of *IRE1* did not alter the ER localisation of Ste6, Ste3, and Cpr2 (Fig. 3a). However, when cultured with cells of the opposite mating type, deletion of *IRE1* delayed the translocation of Ste6 and Ste3, but not Cpr2, from the ER to the cell membrane (Fig. 3b–d). Such delayed translocation was more evident for Ste6-Gfp than Ste3-Gfp. These data suggest that Ire1-mediated ER quality control is likely to be involved in the cellular translocation of the mating pheromone transporter and receptors from the ER to the cell membrane during sexual differentiation. This finding may explain why the *ire1*Δ mutant, with increased *MFα1* expression, showed reduced cell fusion and conjugation tube formation.

### Kar2, an ER-resident molecular chaperone, promotes mating downstream of Ire1

**Figure 4:**
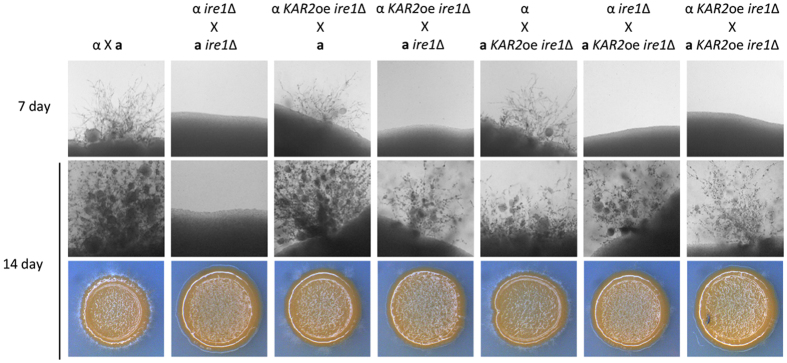
Overexpression of *KAR2* slightly suppressed the filamentation defect of the *ire1*∆ mutant. *C. neoformans* cells were cultured in liquid YPD medium at 30 °C overnight. Then, cells were spotted on V8 medium (pH 5.0) and incubated in the dark for the indicated time period. Representative edges of the mating patches were photographed at 100× magnification. The strains used for the mating assay were as follows: α (H99), **a** (KN99**a**) α *ire1*∆ (YSB552), **a** *ire1*∆ (YSB550), α *KAR2*oe *ire1*∆ (YSB1741), and **a** *KAR2*oe *ire1*∆ (YSB3194).

If Ire1-mediated ER quality control is involved in the mating of *C. neoformans*, then the ER-resident molecular chaperone Kar2 is likely to be involved. Previously, we showed that Kar2 is essential for the viability of *C. neoformans*, and a subset of Ire1-dependent phenotypes, such as the ER and cell wall stress responses, are partially restored by *KAR2* overexpression^19^. According to these observations, we tested whether *KAR2* overexpression can suppress the filamentation defects of the *ire1*Δ mutants. For this purpose, we constructed a *KAR2* overexpression strain in the **a** *ire1*Δ mutant background by replacing the native *KAR2* promoter with the constitutively active H3 promoter. Overexpression of *KAR2* restored filament production in α *ire1*Δ × **a** *ire1*Δ crosses (Fig. 4), albeit not to wild-type levels, indicating that Kar2 plays a role in sexual differentiation as a downstream effector of Ire1. These data further suggest that the Ire1-mediated ER quality control system is required for sexual differentiation.

### Rim101 controls capsule production, but not sexual differentiation, as a downstream effector of Ire1

Because Hxl1, which is a downstream TF of Ire1 in the ER stress response and adaptation, is dispensable for Ire1-dependent regulation of mating in *C. neoformans*, we examined whether other TFs are regulated by Ire1 during mating. In addition to its role in mating regulation, our previous study showed that Ire1 also controls capsule production in an Hxl1-independent manner^18^. Several studies in *S. cerevisiae* have shown that Ire1 interacts with the TFs Ada2 and Gcn5^32^,^33^, and deletion of *ADA2* or *GCN5* causes capsule defects in *C. neoformans*^34^,^35^. Furthermore, the Spt-Ada-Gcn5-acetyltransferase (SAGA) transcriptional coactivator complex regulates differentiation in *S. pombe* and *Fusarium graminearum*^36^,^37^. In line with these observations, perturbation of *ADA2* leads to a mating defect in *C. neoformans*^35^. Rim101 is a direct target of Ada2 in *C. albicans*^38^ and is involved in differentiation and phenotype-switching in *S. cerevisiae* and *C. albicans*^39^,^40^. Moreover, the *rim101*∆ mutant exhibits capsule defects and increased susceptibility to ER stress caused by tunicamycin treatment^41^,^42^.

**Figure 5:**
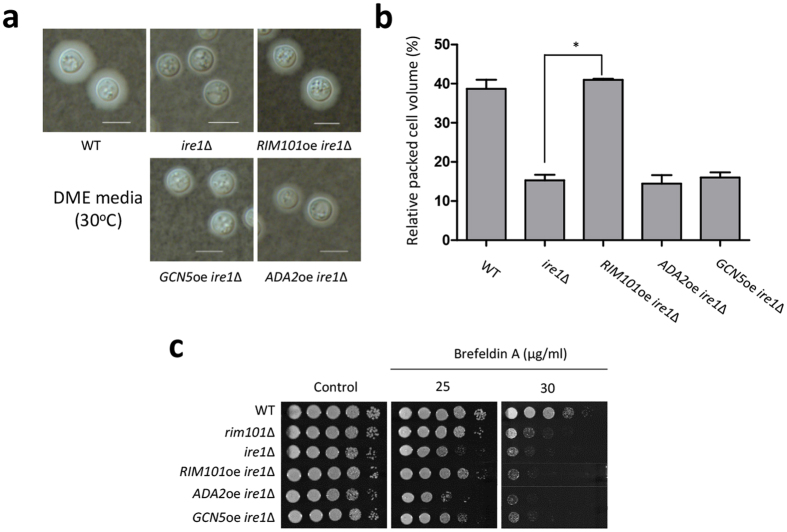
Rim101 contributed to capsule production as a downstream factor of Ire1. (**a,b**) *C. neoformans* strains were cultured in liquid YPD medium for 16 h at 30 °C. Then, the strains were spotted onto solid-agar-based DME medium and incubated at 30 °C for 2 d. After incubation, the cells were scraped and resuspended in PBS and stained with India ink (BACTIDROP^TM^; Remel). For quantitative analysis of capsule production, the relative packed cell volume was measured by calculating the ratio of the length of the packed cell volume to the length of the total volume. The scale bar is 10 μm. (**c**) *Cryptococcus* strains were grown overnight, serially diluted 10-fold (1 to 10^4^), and spotted onto YPD medium containing brefeldin A. The plates were incubated at 30 °C for 2 d and photographed daily. The strains used for the experiment were as follows: WT (H99), *rim101*∆ (YSB1366), *ire1*∆ (YSB552), *RIM101*oe *ire1*∆ (YSB3308), *ADA2*oe *ire1*∆ (YSB3376), and *GCN5*oe *ire1*∆ (YSB3372).

Therefore, to test whether the SAGA complex regulates Hxl1-independent Ire1 functions, such as capsule formation and mating, we constructed *ADA2-*, *GCN5*-, and *RIM101-*overexpressing strains in the *ire1*Δ mutant background. First, we determined whether *RIM101*, *ADA2*, or *GCN5* overexpression could restore defective capsule production in the *ire1*Δ mutant (Fig. 5a,b [quantitative measurement by Cryptocrit]). Overexpression of *RIM101* restored capsule production in the *ire1*Δ mutant (Fig. 5a,b). However, capsule biosynthesis was not restored in the *ADA2-* or *GCN5-*overexpressing strains.

The capsular polysaccharides glucuronoxylomannan and galactoxylomannan are secreted from *C. neoformans* into the extracellular environment through vesicle trafficking, and then they bind to the cell wall^43^. To elucidate the role of Ire1 in cellular secretion, we monitored the resistance of the *ire1*∆ mutant to brefeldin A (BFA), which is an inhibitor of vesicle trafficking. Notably, the *ire1*Δ mutant showed a growth defect when exposed to BFA (Fig. 5c). Overexpression of *RIM101*, but not *ADA2* or *GCN5*, partially suppressed the growth defect of the *ire1*∆ mutant, suggesting that Rim101, but not Ada2 or Gcn5, is involved in Ire1-medicated vesicle-trafficking in *C. neoformans*.

Next, we tested whether overexpression of *ADA2*, *GCN5,* or *RIM101* suppresses the filamentation defects of the *ire1*∆ mutants. However, constitutive overexpression of *RIM101, ADA2,* or *GCN5* did not restore filament production in bilateral α *ire1*Δ × **a** *ire1*Δ mutants (Supplementary Figure S2). Taken together, these data suggest that Rim101 is likely a TF that is downstream of Ire1 in capsule production, but not sexual differentiation.

### Ire1 regulates unisexual differentiation

**Figure 6:**
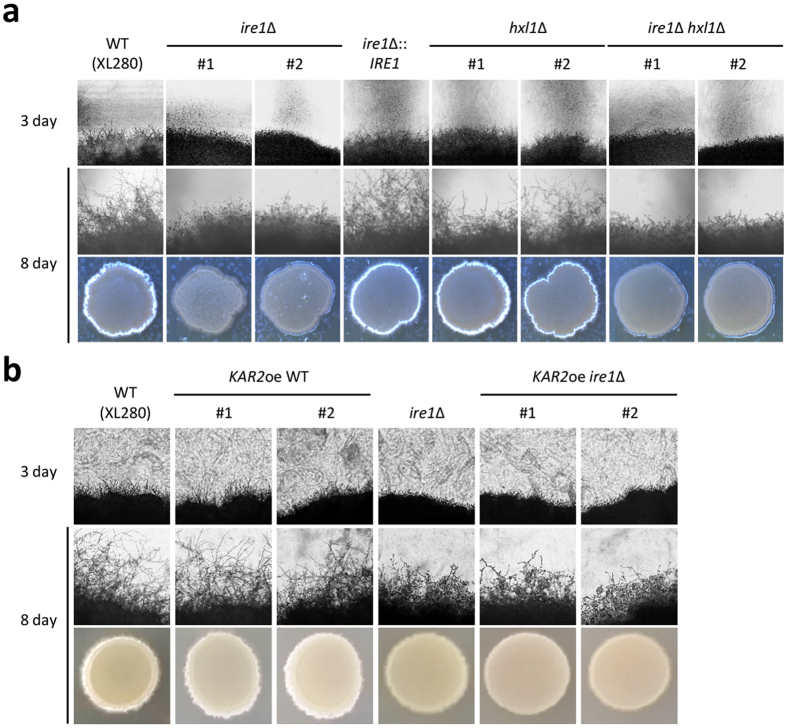
Ire1 regulates same-sex mating in a Kar2-independent manner. (**a**) Ire1 regulates same-sex mating in an Hxl1-independent manner. Strains were cultured in liquid YPD medium at 30 °C overnight. Then, cells were spotted on V8 medium (pH 7.0) and incubated in the dark at room temperature for the indicated time period. The cells and periphery of colonies were visualised using a microscope equipped with a digital camera. (**b**) *KAR2* overexpression did not suppress the mating defect of the XL280 *ire1*∆ mutant. Representative edges of the mating patches were photographed at 100× magnification. The strains used for the mating assays were as follows: XL280, α *ire1*∆ (YSB2144 and YSB2145, labelled 1 and 2, respectively), α *hxl1*∆ (YSB2147 and YSB2148, labelled 1 and 2, respectively), α *ire1*∆ *hxl1*∆ (KW345 and 346, labelled 1 and 2, respectively), α *ire1*∆:: *IRE1* (YSB3596), α *KAR2*oe (YSB3492 and YSB3493), and α *KAR2*oe *ire1*∆ (YSB3499 and YSB3500, labelled 1 and 2, respectively).

In *C. neoformans*, mating pheromone plays dual roles in paracrine and autocrine signalling^44^. For its paracrine function, this pheromone stimulates cell-cell fusion and conjugation with cells of the opposite mating type. For its autocrine role, this pheromone activates filamentation and sporulation of *MAT*α cells during nutrient starvation. Given the function of Ire1 in the sexual differentiation of *C. neoformans*, we questioned whether Ire1 also regulates same-sex mating. To answer this question, we constructed UPR mutants in the background of the same-sex mating tester strain XL280 (serotype D), which shows robust unisexual reproduction^16^, and conducted a same-sex mating assay. The XL280 *ire1*Δ mutant showed defects in same-sex mating when compared to the XL280 strain and the complemented XL280 *ire1*Δ mutant strain (Fig. 6a). However, *hxl1*Δ mutants showed wild-type levels of same-sex mating (Fig. 6a). Moreover, *ire1*Δ *hxl1*Δ double mutants exhibited filamentation defects similar to the *ire1*Δ mutant (Fig. 6a). These data indicated that Ire1 also regulates same-sex mating in an Hxl1-independent manner.

The finding that overexpression of *KAR2* suppressed the sexual mating defect of the *ire1*Δ mutant in the serotype A strain prompted us to determine whether Kar2 has similar functions in same-sex mating. For this purpose, we constructed constitutive *KAR2*-overexpressing strains in the XL280 *ire1*Δ mutant background. Unexpectedly, overexpression of *KAR2* did not restore the same-sex mating ability of the *ire1*Δ mutant in the XL280 strain (Fig. 6b). This result suggests that Ire1 controls the same-sex mating response in a Kar2-independent manner.

## Discussion

Over the past few decades, the roles of the UPR pathway have been extensively characterised in eukaryotic organisms ranging from yeasts to humans. Many studies have demonstrated that the UPR pathway consists of Ire1 kinase/endoribonuclease and the downstream factor Hac1/Xbp1 and plays pivotal roles in sensing, responding, and counteracting ER stress triggered by accumulated unfolded or misfolded proteins. However, it is evident that Ire1 has unique functions in diverse stress responses and in the differentiation of eukaryotic cells, including plants, insects, and fungi^45^,^46^,^47^. The UPR pathway in the human fungal pathogen *C. neoformans* contains the evolutionarily conserved Ire1 kinase, but harbours a unique downstream factor, Hxl1^18^. As in other eukaryotes, Ire1 has both Hxl1-dependent and -independent functions in *C. neoformans*^18^. In the present study, we showed that *C. neoformans* Ire1 controls opposite- and same-sex mating in an Hxl1-independent manner and capsule production in an Hxl1-independent, but Rim101-dependent manner, suggesting that the UPR pathway modulates differentiation and developmental processes in basidiomycetous fungi.

Although the UPR pathway plays evolutionarily conserved roles in ER and cell wall stress responses in fungi, it performs distinct functions in sexual differentiation. In *S. cerevisiae*, diploid *ire1*∆ and *hxl1*∆ mutants show enhanced pseudohyphal growth, suggesting that both UPR components suppress filamentous growth. In contrast, they have distinct roles in meiosis. Hac1 negatively regulates the expression of early meiotic genes, including *IME2*, *HOP1*, and *SPO13*, whereas Ire1 controls the expression of *IME1*, a positive regulator of meiosis^5^. Schroder *et al.* reported that Hac1 represses differentiation by recruiting the Rpd3-Sin3 HDAC as a negative regulator of early meiosis genes^48^. However, the roles of the UPR pathway in *Cryptococcus* appear to be different from those in *S. cerevisiae*. Ire1, but not Hxl1, performs critical functions in the sexual mating of serotypes A and D by positively regulating cell-cell fusion and conjugation tube formation. Moreover, perturbation of *IRE1* decreased the efficiency of α-α same-sex mating. In line with this result, in the basidiomycetes *U. maydis*, Ire1, but not Cib1, reduces *b* mating-type-dependent filament formation^6^. Thus, in the UPR pathway, the roles of basidiomycetes Ire1 in differentiation are evolutionarily distinct from those of the in ascomycetes *S. cerevisiae* Ire1.

The signalling pathways involved in the sexual differentiation of *C. neoformans* have been widely studied. The Cpk1 MAPK pathway controls both α-**a** opposite mating and α-α same-sex mating in *C. neoformans*^21^,^49^,^50^,^51^,^52^,^53^. Furthermore, the Ras signalling pathway positively regulates the mating response by changing mating pheromone expression^26^,^54^. In contrast to its positive role in cell-cell fusion and conjugation tube formation, Ire1 suppresses pheromone gene expression, suggesting that Ire1 plays both negative and positive roles at the early stage of mating. Notably, the increased pheromone gene expression in the *ire1*∆ mutant was significantly decreased by deletion of *CPK1* or *RAS1*, implying that Ire1 controls the mating response in a Cpk1 MAPK- and Ras signalling pathway-dependent manner. Supporting this notion, a bilateral cross with a *crg1*Δ *ire1*Δ double mutant yielded a decreased mating response. The detailed mechanism underlying the crosstalk among the Ire1-, Cpk1-, and Ras1-mediated mating pathways should be further investigated in future studies.

One unexpected finding in this study is that in *C. neoformans,* the pheromone transporter Ste6 and the pheromone receptor Ste3/Cprα transition from the ER to the plasma membrane during mating. In *Cryptococcus,* Ste6 and Ste3 are mainly localised to the ER under basal conditions in the absence of a mating partner, but these proteins re-localise to the plasma membrane after sensing the presence of the opposite mating type. Some researchers have reported that Cpr2 is evenly distributed on the plasma membrane during the mating response, which is similar to our results^30^. However, in *S. cerevisiae*, Ste2, which is the α-factor receptor, is evenly distributed on the plasma membrane even under basal conditions without a mating partner. After exposure to **a** pheromone, Ste2 is internalised and forms polarised receptors toward the nearest mating partner^55^. Therefore, it seems that mating-induced cellular re-localisation of mating components may vary among fungi. In addition to this post-translational control, in *C. neoformans*, the expression levels of *STE6*, *STE3*, and *CPR2* are strongly induced at the initial stage of the mating response and then return to basal levels^30^,^31^. Increased production of the mating pheromone transporter and receptors during mating may affect ER burden because such membrane proteins should be properly folded in the ER. Therefore, these studies indicate that transcriptional and post-translational control mechanisms, including the changes in the localisation of mating components, are crucial for the mating response in *C. neoformans*. The regulatory mechanism underlying the mating-induced re-localisation of pheromone transporters and receptors in *C. neoformans* should be elucidated in future studies. This re-localisation could be under control of a positive feedback loop, because a certain level of pheromone receptor should be present on the plasma membrane to recognise the pheromone secreted by the opposite mating type. Then, the Cpk1-pheromone responsive MAPK pathway may trigger a transition in cellular localisation by engaging in crosstalk with the Ire1-dependent pathway.

The notable finding in this study is that protein quality control is involved in the mating response of *C. neoformans*. Several lines of evidence provided by this and other studies support this hypothesis. Firstly, we demonstrated that Kar2, an ER-resident molecular chaperone downstream of Ire1, is involved in the mating response. In agreement with this finding, overexpression of *KAR2* partially recovered the mating efficiency of the *ire1*Δ mutants. Secondly, in *S. cerevisiae*, expression of Ste2-3, a mutant pheromone receptor that is defective in targeting to the plasma membrane, results in a mating defect. Deletion of *SOP4*, which encodes an ER membrane protein, increases the proper delivery of Ste2-3 to the plasma membrane, resulting in enhanced mating efficiency^27^. Thirdly, the ER-associated degradation (ERAD) pathway, which is one of the major protein quality control systems and involves the ubiquitin-proteasome system, affects the mating response. Deletion of *UBP5*, which encodes a deubiquitinating enzyme, or *FBP1*, which encodes a ubiquitin ligase, causes a severe mating defect in *C. neoformans*^56^,^57^. Furthermore, in *S. cerevisiae*, Ste6, a mating pheromone transporter, undergoes ubiquitination-mediated protein quality control in the ER^28^. In line with this observation, we demonstrated that mating-induced localisation of Ste6 from the ER to the cell membrane is delayed by deletion of *IRE1*. As is the case for Ste6, we also found that mating-induced localisation of a major pheromone-sensing G protein-coupled receptor, Ste3, is also delayed by *IRE1* deletion.

If Ire1 controls the sexual and unisexual differentiation of *C. neoformans* in an Hxl1-independent manner, the next obvious question is what other TF(s) function downstream of Ire1 in mating. To identify such a TF, we turned our attention to other Ire1-dependent, Hxl1-independent phenotypes. Our previous study showed that Ire1 controls capsule production in an Hxl1-independent manner; however, the mechanism underlying Ire1-mediated capsule production has not been fully characterised^18^. Based on these findings, Ada2, which is part of the SAGA complex, was our best candidate for the following reasons: 1) In *C. neoformans*, Ada2 is involved in both capsule formation and mating^35^ and 2) in *S. cerevisiae*, Ire1 interacts with Ada2 and Gcn5^32^,^33^. Nevertheless, our data showed that Ada2 is not downstream of Ire1. Overexpression of *ADA2* did not restore the mating and capsule defects of the *ire1*∆ mutants. Similarly, overexpression of *GCN5*, which is another SAGA complex component, did not restore the *ire1*∆ mutant phenotypes. Notably, our study revealed that Rim101, which is a direct target of Ada2 in *C. albicans*^38^, may be the second TF downstream of Ire1, at least in the control of secretion or attachment of the polysaccharide capsule. In line with this result, *RIM101* overexpression in the *ire1*∆ mutant rendered cells resistant to brefeldin A, which is an inhibitor of the anterograde transport of proteins between the ER and Golgi, when compared to the resistance of the *ire1*∆ mutant. Moreover, various studies revealed that Rim101 regulates the expression of genes related to cell wall remodelling for capsule attachment rather than capsule production^42^,^58^. Although both the *ire1*∆ and *hxl1*∆ mutants had cell wall defects, the expression levels of *CHS2* (chitin biosynthesis) are regulated by Ire1, but not Hxl1, during ER stress^18^. Nevertheless, Rim101 is not likely to be involved in the Ire1-dependent mating process, because overexpression of *RIM101* could not restore the mating defect in the *ire1*∆ mutant. Therefore, the Ire1-regulated TF that modulates sexual differentiation remains to be discovered.

**Figure 7:**
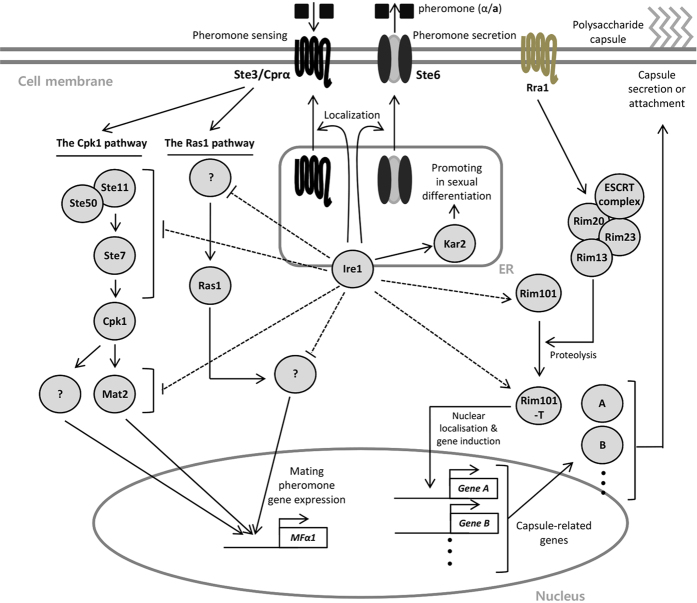
The proposed model of Ire1 function in capsule production and differentiation of *C. neoformans*. Overexpressed Rim101 restores capsule production in the *ire1*∆ mutant by enhancing secretion. Ire1 influences the localisation of the mating pheromone transporter (Ste6) and receptor (Ste3/Cprα) during mating. The molecular chaperone Kar2 partially affects sexual-mating efficiency as a downstream effector of Ire1. Ire1 negatively regulates the gene expression of mating pheromone (*MFα1*) in a Cpk1- and Ras1-dependent manner. ERCRT complex contains Vps23, Vps25, and Snf7. Rim101-T represents Rim101 transcription factor.

In conclusion, we demonstrated that the UPR pathway modulates the sexual and unisexual differentiation of *C. neoformans* in an Hxl1-independent manner and that Rim101 is a novel TF downstream of Ire1 in capsule production (summarised in Fig. 7). Therefore, our present and previous studies^18^ clearly demonstrate that the UPR pathway is essential for the growth, differentiation, and proliferation of *C. neoformans* in both natural and host environments.

## Methods

### Strains and growth conditions

The *C. neoformans* strains used in this study are listed in Supplementary Table S1. V8 medium (pH 5.0 for serotype A and pH 7.0 for serotype D; Campbell, Camden, NJ) which was used for mating and DME medium which was used for capsule production were prepared as previously described^59^,^60^.

### Deletion of *IRE1* and *HXL1*

The *ire1*Δ and *hxl1*Δ mutants were constructed in the *C. neoformans* serotype A KN99**a**, *crg1*∆, serotype D JEC21 and JEC20, and XL280 strain. To construct *ire1*∆ and *hxl1*∆ mutants of the serotype A and D strains, we used primers to amplify the 5′- and 3′-flanking regions of the serotype A and D *IRE1* and *HXL1* genes (Supplementary Table S2). In the first round of PCR, the 5′- and 3′-flanking regions of *IRE1* and *HXL1* were amplified using primers L1 and L2 and primers R1 and R2, respectively, from genomic DNA from the H99 or JEC21 strains. To amplify the dominant selection marker (NAT^R^ or NEO^R^), primers M13Fe and M13Re were used. In the second round of PCR, *IRE1* or *HXL1* gene deletion cassettes were constructed by double-joint PCR. To amplify the 5′- and 3′-regions of the NAT-split markers, primers M13Fe and B1455 and primers M13Re and B1454 were used, respectively. To amplify the 5′- and 3′-regions of the NEO-split markers, primers M13Fe and B1887 and primers M13Re and B1886 were used, respectively^61^. The disruption cassettes were introduced into the *C. neoformans* serotype A strains KN99**a** and *crg1*∆ strains and the serotype D strains JEC21, JEC20, and XL280 through biolistic transformation, as previously described^62^. Stable nourseothricin- or neomycin-resistant transformants were initially screened by diagnostic PCR, and then the genotype of the positive transformants was confirmed by Southern blot analysis, as previously described^63^ (Supplementary Figures S3–S5).

The *ire1*∆ *hxl1*∆ double mutants were generated by introducing the *HXL1*-deletion cassette into the serotype A α *ire1*∆ (YSB552) and **a** *ire1*∆ (YSB550) mutants and the XL280 strain *ire1*∆ (YSB2145) by biolistic transformation. Stable transformants selected on YPD medium containing nourseothricin or neomycin were initially screened by diagnostic PCR. Then, their genotypes were confirmed by Southern blot analysis (Supplementary Figures S3 and S5).

### Construction of constitutively *KAR2*-, *ADA2*-, *RIM101*-, and *GCN5*-overexpressing strains

To construct these strains, we inserted the histone H3 gene promoter along with a selectable marker upstream of the ATG start codon of each target gene. To construct strains that constitutively overexpress *KAR2*, we generated a P*H3*:*KAR2* cassette, as previously described^19^. Then, the P*H3*:*KAR2* cassette was introduced into the wild-type *MAT***a** KN99**a** and *ire1*Δ mutant (YSB550) strains (Supplementary Figure S6). To construct XL280 strains and *ire1*Δ mutants (YSB2144 and YSB2145) that constitutively overexpress *KAR2*, the P*H3:KAR2* cassette was generated as follows. In the first round PCR, the 5′-flanking region of *KAR2* was amplified with primers B6633 and B6634, the 5′-exon region of *KAR2* was amplified with primers B6635 and B6644, and the NEO-H3 promoter fragments were amplified with primers B4017 and B4018 from the plasmid pNEO-H3 promoter. Next, the left fusion fragment was amplified with primer pairs B6633/B1887 from the first-round PCR products containing the 5′-flanking region of *KAR2* and the NEO-H3 promoter fragments. The right-hand fusion fragment was amplified with the primer pair B6644/B1886 from the first-round PCR products containing the 5′-exon region of *KAR2* and the NEO-H3 promoter fragments. Then, the two DJ-PCR products were mixed and biolistically delivered into the XL280 and *ire1*Δ mutant (YSB2144 and YSB2145) strains (Supplementary Figure S7).

To construct constitutively *RIM101*-overexpressing strains in the serotype A H99 strain background, we generated a P*H3:RIM101* cassette as follows. In the first-round PCR, the 5′-flanking region of *RIM101* was amplified with primers B2982 and B6201, the 5′-exon region of *RIM101* was amplified with primers B6202 and B6203, and the *NEO-H3* promoter fragment was amplified with primers B4017 and B4018 from the plasmid pNEO-H3 promoter. Next, two separate DJ-PCR products were amplified as follows. The left fusion fragment was amplified with primers B2982 and B1887 from a template containing the 5′-flanking region of *RIM101* and the *NEO-H3* promoter fragments. The right-hand fusion fragment was amplified with primers B6203 and B1886 from a template containing the 5′-exon region of *RIM101* and the *NEO-H3* promoter fragments. Then, the two DJ-PCR products were mixed and biolistically delivered into the *ire1*Δ mutant (YSB552) strain (Supplementary Figure S8).

Constitutively *ADA2-* or *GCN5-*overexpressing strains were constructed in the serotype A H99 background using a P*H3:ADA2* or P*H3:GCN5* cassette as follows. In the first round PCR, the 5′-flanking regions of *ADA2* or *GCN5* (amplified with primer pairs B2183/B6399 or B6402/B6403, respectively), the 5′-exon region of *ADA2* or *GCN5* (amplified with primer pairs B6400/B2187 or B6404/B6405, respectively), and the NEO-H3 promoter (amplified with primers B4017 and B4018) were amplified using the pNEO-H3 promoter plasmid as a template. Then, the left fusion fragments of the 5′-flanking region of *ADA2* and the NEO-H3 promoter fragments or the 5′-flanking region of *GCN5* and the NEO-H3 promoter fragments were amplified with primer pairs B2183/B1887 or B6401/B1887, respectively. The right fusion fragments of the 5′-exon region of *ADA2* and the NEO-H3 promoter fragments or the 5′-exon region of *GCN5* and the NEO-H3 promoter fragments were amplified with primer pairs B2187/B1886 or B6405/B1886, respectively. Next, the two DJ-PCR products were combined and biolistically delivered into the *ire1*Δ mutant (YSB552).

We confirmed the correct insertion of the H3 promoter by Southern blot analysis and monitored the basal expression levels of the target genes by northern blot analysis (Supplementary Figure S8).

### Construction of the *STE6-GFP*, *STE3-GFP*, and *CPR2-GFP* strains

To construct the *STE6-GFP* strain, we generated three PCR products as follows. In the first-round PCR, the 3′-exon region of *STE6* (CNAG_03600) was amplified with primers B5661 and B5662 using H99 genomic DNA as a template, the *GFP-HOG1*ter*-NEO* fragment was amplified with primers B354 and B5665 using plasmid pNEO-GFPht as a template, and the 3′-flanking region of *STE6* was amplified with primers B5663 and B5664 using H99 genomic DNA as a template. Next, two separate DJ-PCR products were amplified as follows. Primer pair B5661/B1886 was used to amplify a fusion fragment from a template containing the 3′-exon region of *STE6* and the *GFP-HOG1*ter*-NEO* fragment. Primers B5664 and B1887 were used to amplify a fusion fragment from a template containing the *GFP-HOG1*ter*-NEO* fragment and the 3′-flanking region of *STE6*. Then, the two DJ-PCR products were combined and biolistically delivered into the H99 strain.

To construct the *STE3-GFP* and *CPR2-GFP* strains, three PCR products were separately generated in the first round as described above: (1) The 3′-exon region of *STE3* (CNAG_06808) was amplified with primers B5897 and B5898 and the 3′-exon region of *CPR2* (CNAG_03938) was amplified with primers B5943 and B5902, (2) the *GFP-HOG1*ter*-NEO* fragment was amplified with primers B354 and B5665 using pNEO-GFPht as a template, and (3) the 3′-flanking region of *STE3* was amplified with primers B5899 and B5900 or the 3′-flanking region of *CPR2* was amplified with primers B5903 and B5904. In the second round of PCR, a fusion fragment that contains the 3′-exon regions of *STE3* or *CPR2* and a 5′ split region of the *GFP-HOG1ter-NEO* fragment was amplified with primer pairs B1886/B5897 and B1886/B5943, respectively, from the first-round PCR products (1) and (2) as the templates. Another fusion fragment that contains the 3′-flanking regions of *STE3* or *CPR2* and the 3′ split region of the *GFP-HOG1ter-NEO* fragment were generated with primer pairs B1887/B5900 or B1887/B5904, respectively, from first-round PCR products (2) and (3) as the templates. Next, the two DJ-PCR cassettes were combined and biolistically transfected into the H99 strain.

Targeted integration of the *STE6-GFP*, *STE3-GFP* or *CPR2-GFP* allele into the native locus of each gene was confirmed by diagnostic PCR and Southern blot analysis (Supplementary Figure S9).

For ER staining, cells from each sample were fixed as previously described^64^ and treated with an ER tracker (Invitrogen, cat. # E34250). The cells were visualised by fluorescence microscopy (Nikon Eclipse Ti microscope).

### Capsule production assay

*C. neoformans* strains were cultured in liquid YPD medium at 30 °C for 16 h, and then spotted onto DME medium and incubated at 30 °C for 2 d. After incubation, the capsules were stained with India ink (BACTIDROP^TM^, Remel) and visualised with an Olympus BX51 microscope equipped with a SPOT insight digital camera. For quantitative analysis of the capsules, the relative packed cell volume was measured with haematocrit capillary tubes as previously described^63^.

### Mating, cell fusion, and confrontation assays

*C. neoformans* strains were cultured in 2 mL of YPD medium at 30 °C for 16 h. After cell counting, equal numbers of *MAT*α and *MAT***a** cells were mixed, spotted onto V8 mating medium (pH 5 for serotype A and pH 7 for serotype D), and further incubated in the dark at room temperature for 1–2 weeks. Filamentous growth was monitored and photographed using a microscope equipped with a SPOT Insight digital camera (Diagnostic Instrument, Inc.). Cell fusion and confrontation assays were performed as previously described^59^,^63^.

### Expression analysis by northern blotting and quantitative RT-PCR

To monitor the expression levels of *MFα1*, we performed northern blot analysis and quantitative real-time PCR. For the northern blot, the membrane was hybridised with a radioactively labelled probe using gene-specific primers, as previously described^63^. For quantitative RT-PCR analysis, the expression levels of genes were measured using gene-specific primers on a MyiQ2 Real-Time PCR detection system (Bio-Rad).

## Additional Information

**How to cite this article**: Jung, K.-W. *et al.* Unique roles of the unfolded protein response pathway in fungal development and differentiation. *Sci. Rep.* **6**, 33413; doi: 10.1038/srep33413 (2016).

## Supplementary Material

Supplementary Information
