# Supplementary material for: Unique roles of the unfolded protein response pathway in fungal development and differentiation
Source: Sci Rep. 2016 Sep 15;6:33413. doi: 10.1038/srep33413 (PMC5024300; doi:10.1038/srep33413)
Supplement: Supplementary Information [file srep33413-s1.pdf]

Supplementary Information

Unique roles of the unfolded protein response pathway in fungal development and differentiation

Kwang-Woo Jung, Yee-Seul So, & Yong-Sun Bahn*

Contents

- **Supplementary Figure S1**
- **Supplementary Figure S2**
- **Supplementary Figure S3**
- **Supplementary Figure S4**
- **Supplementary Figure S5**
- **Supplementary Figure S6**
- **Supplementary Figure S7**
- **Supplementary Figure S8**
- **Supplementary Figure S9**
- **Supplementary Table S1**
- **Supplementary Table S2**

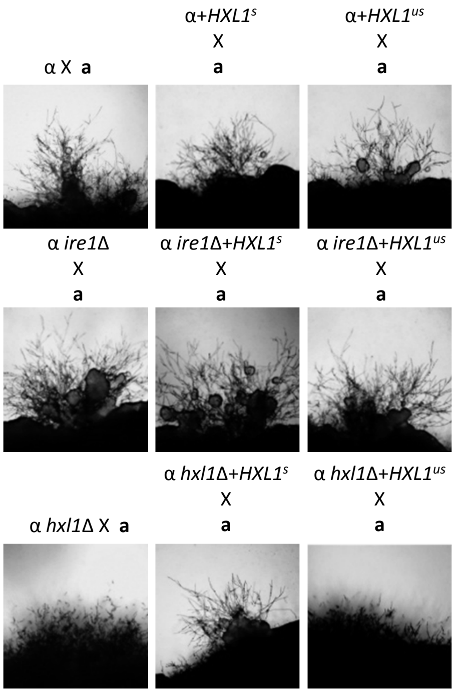

Supplementary Figure S1. Overactivation of Hxl1 does not affect sexual differentiation in *C. neoformans*. Each *Cryptococcus* strain was cultured in the liquid YPD medium at 30°C overnight. Next, equal concentrations of *MAT*α and *MAT***a** strains were mixed and spotted onto a V8 mating medium (pH 5). Serotype A strains were co-cultured on the V8 medium (pH 5.0) for 10 d. Strain information: α (H99), **a** (KN99**a**), α *ire1*Δ mutant (YSB552), α *hxl1*Δ mutant (YSB723), α+*HXL1*^{s} (YSB742), α+*HXL1*^{us} (YSB737), α *ire1*Δ+*HXL1*^{s} (YSB1127), α *ire1*Δ+*HXL1*^{us} (YSB1126), α *hxl1*Δ+*HXL1*^{s} (YSB1225), and α *hxl1*Δ+*HXL1*^{us} (YSB762).

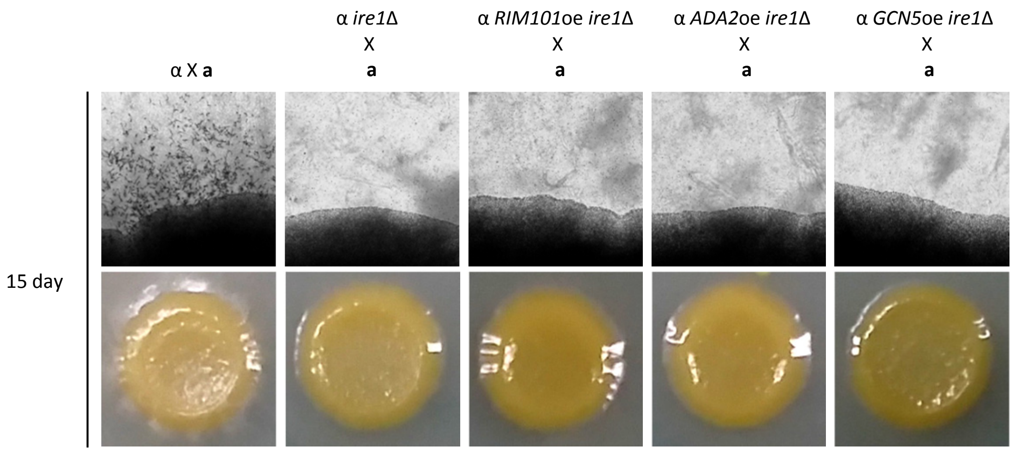

Supplementary Figure S2. Overexpression of *ADA2*, *RIM101*, or *GCN5* did not suppress the mating defect of the *ire1*Δ mutant. Serotype A *MAT*α and *MAT***a** strains were grown in the liquid YPD medium at 30°C for 16 h. After cell counting, *MAT*α and *MAT***a** strains were co-cultured on the V8 medium (pH 5.0) for 15 d at room temperature in the dark: α (H99) × **a** (KN99**a**), α *ire1*Δ (YSB552) × **a** *ire1*Δ (YSB550), α *RIM101*oe *ire1*Δ (YSB3308) × **a** *ire1*Δ (YSB550), α *ADA2*oe *ire1*Δ (YSB3376) × **a** *ire1*Δ (YSB550), and α *GCN5oe ire1*Δ (YSB3372) × **a** *ire1*Δ (YSB550). Representative edges of the mating patches were photographed at ×100 magnification.

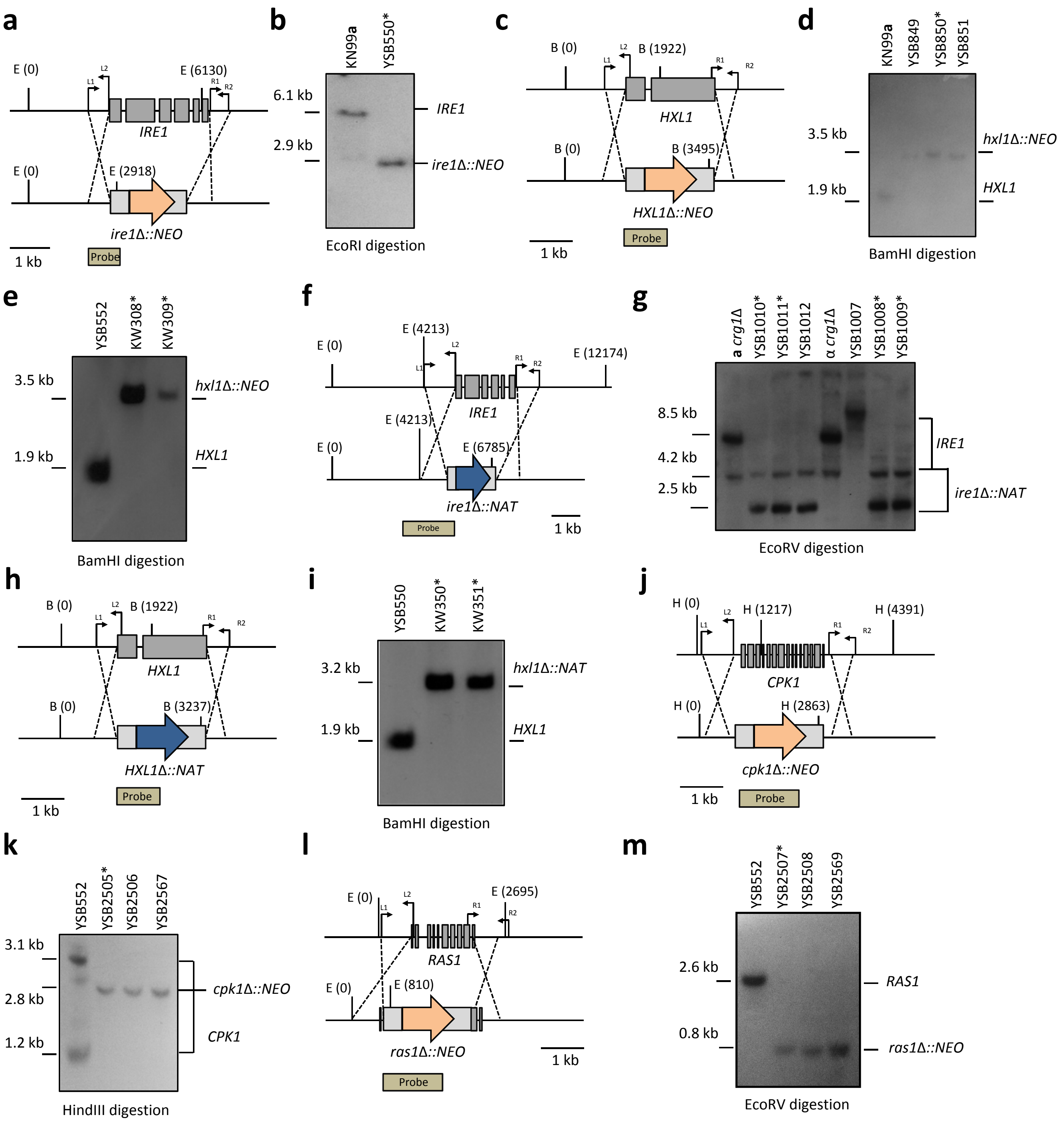

Supplementary Figure S3. Disruption of *C. neoformans IRE1* and *HXL1* in serotype A *MAT*a KN99a, *ire1*Δ mutants and *crg1*Δ strains and disruption of *CPK1* and *RAS1* in the *ire1*Δ mutants. (a,c,f, h, j, and l) A diagram of disruption of the *IRE1* gene, *HXL1* gene, *CPK1* gene, and *RAS1* gene in the serotype A (*MAT***a**) KN99**a** strain, *ire1*Δ mutants (YSB552 and YSB550), *crg1*Δ mutants (H99 crg1 and PPW196). (b, d, e, g, i, k, and m) The correct gene disruption was verified by Southern blot analysis using genomic DNAs digested with the indicated restriction enzyme. Strains marked with asterisk were used in this study.

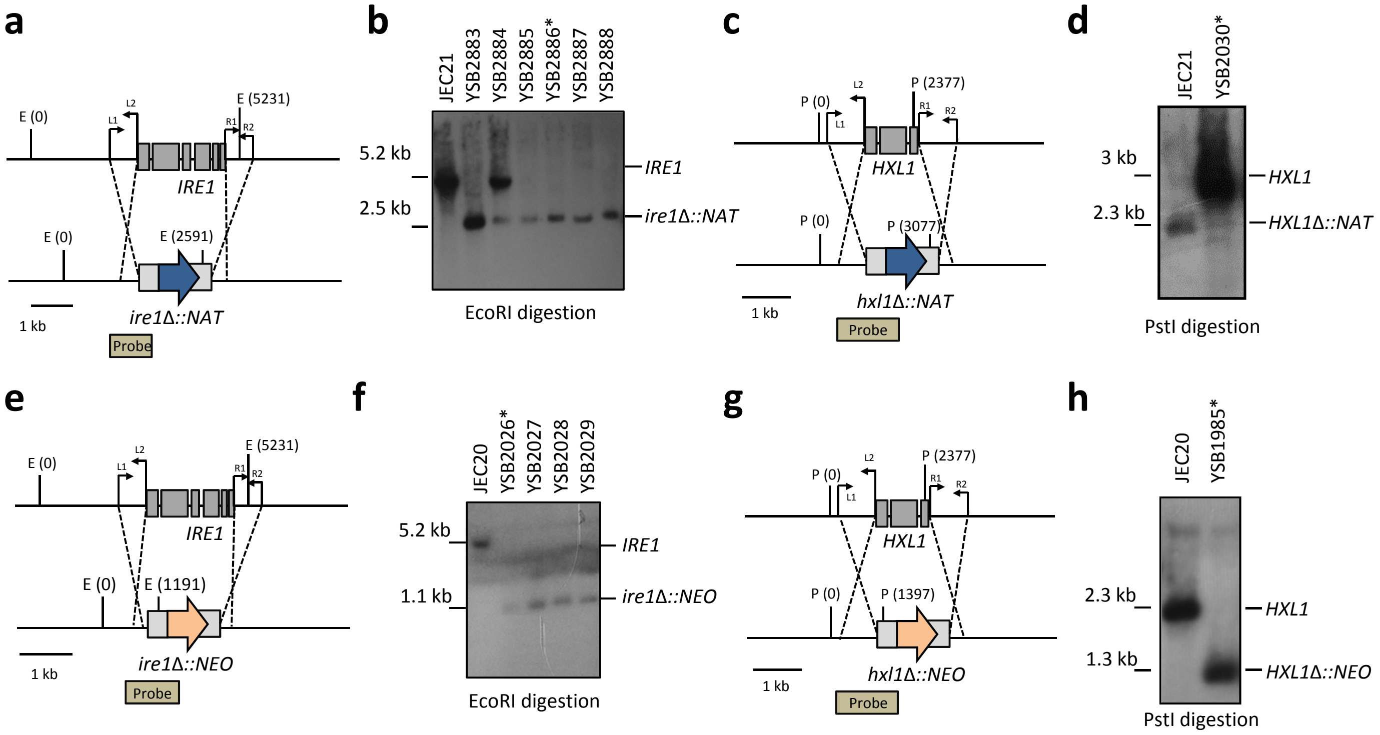

Supplementary Figure S4. Disruption of *C. neoformans IRE1* and *HXL1* in serotype D JEC21 and JEC20 strains. (a, c, e, and g) A diagram of disruption of the *IRE1* gene and *HXL1* gene in serotype D JEC21 (*MAT*α) strain and JEC20 (*MAT***a**). (b, d, f, and h) The correct gene disruption was verified by Southern blot analysis using genomic DNAs digested with the indicated restriction enzyme. Strains marked with asterisk were used in this study.

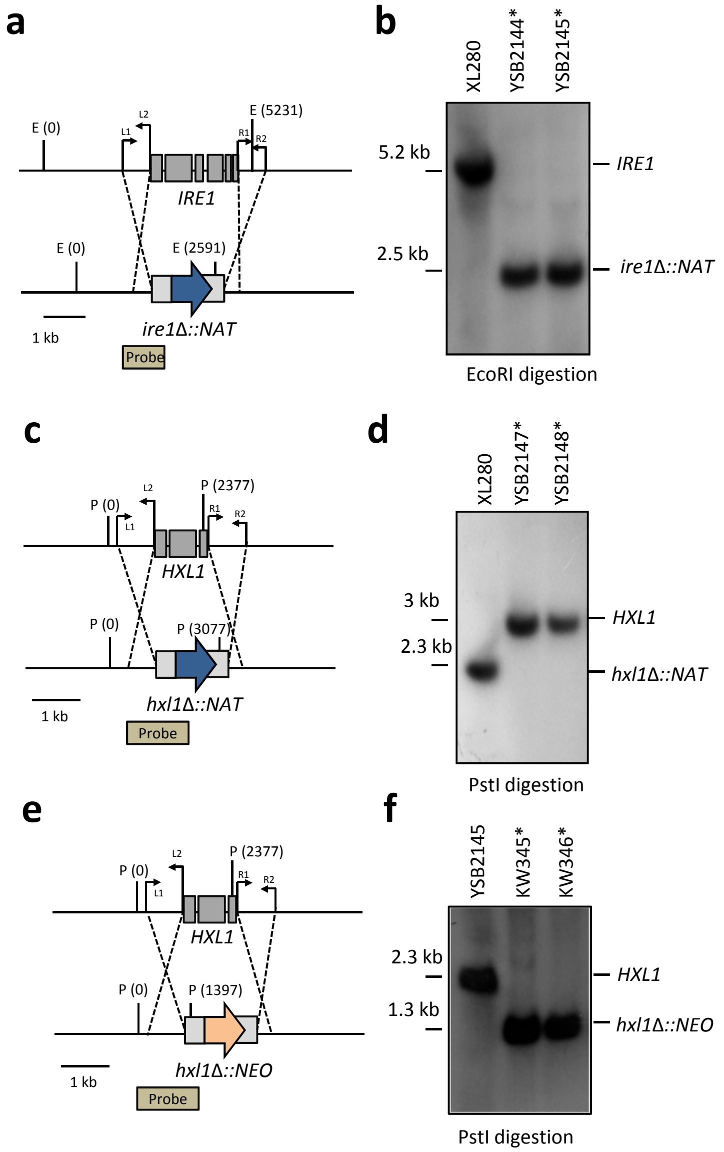

Supplementary Figure S5. Deletion of *C. neoformans IRE1* and *HXL1* in the XL280 strain and *ire1*Δ mutant. (a, c, and e) A diagram of disruption of the *IRE1* gene and *HXL1* gene in the XL280 strain. (b, d, and f) The correct gene disruption was verified by Southern blot analysis using genomic DNAs digested with the indicated restriction enzyme. Strains marked with asterisk were used in this study.

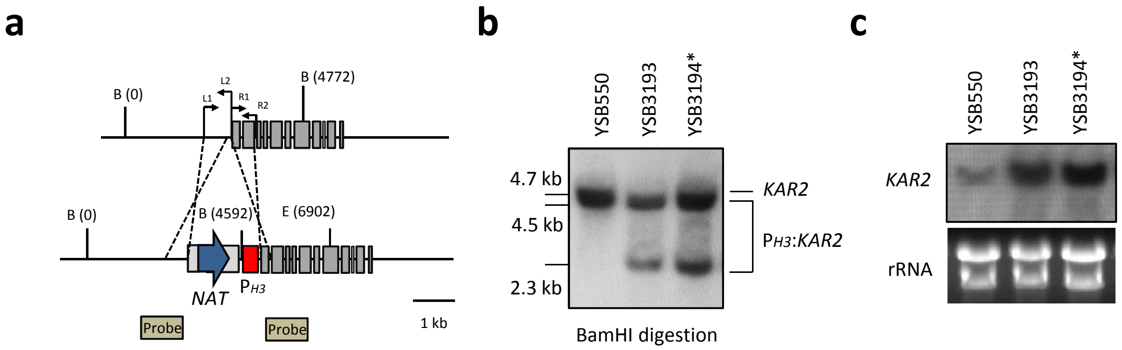

Supplementary Figure S6. Construction of the constitutively *KAR2*-overexpressing strains in *C. neoformans*. (a) The strategy for construction of the P*H3:KAR2* strain containing the NAT resistance marker (NAT^R) and the histone H3 gene promoter (P*H3*). (b) The correct genotype of the P*H3:KAR2* strain in the background of the *ire1*Δ mutant was confirmed by Southern blot analysis using genomic DNAs digested with the restriction enzyme BamHI. The membrane was hybridised with a *KAR2*-specific probe, washed, and developed. (c) Northern blot analysis for measurement of *KAR2* expression in P*H3:KAR2* strains. Data from ethidium bromide staining of rRNA was used for loading control. Strains marked with asterisk were used in this study.

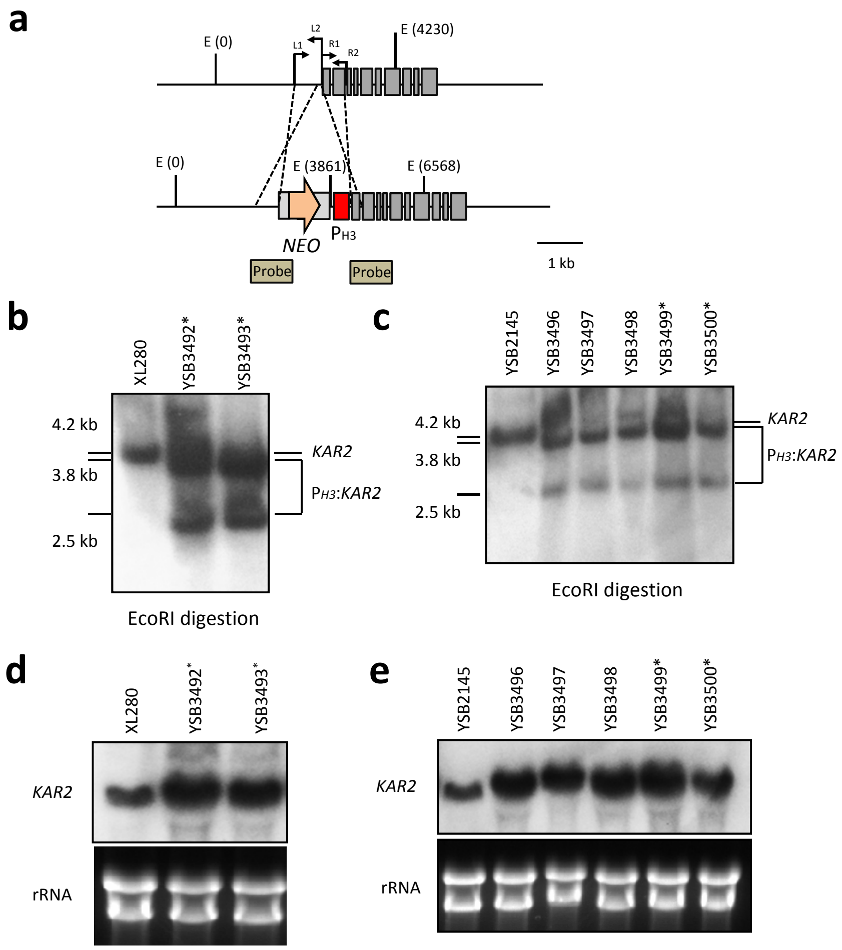

Supplementary Figure S7. Construction of the constitutively *KAR2*-overexpressing strains from the XL280 and *ire1*Δ mutant. (a) A diagram of construction of the constitutively *KAR2*-overexpressing strains using H3 promoter. (b and c) The correct genotype of the P*H3:KAR2* strain in each background strain (XL280: YSB3492 and YSB3493 and *ire1*Δ mutant: YSB3499 and YSB3500) was confirmed by Southern blot analysis using genomic DNAs digested with the restriction enzyme EcoRI. The membrane was hybridised with a *KAR2*-specific probe, washed, and developed. (d and e) Northern blot analysis for measurement of *KAR2* expression in P*H3:KAR2* strains. Data from ethidium bromide staining of rRNA was used for loading control. Strains marked with asterisk were used in this study.

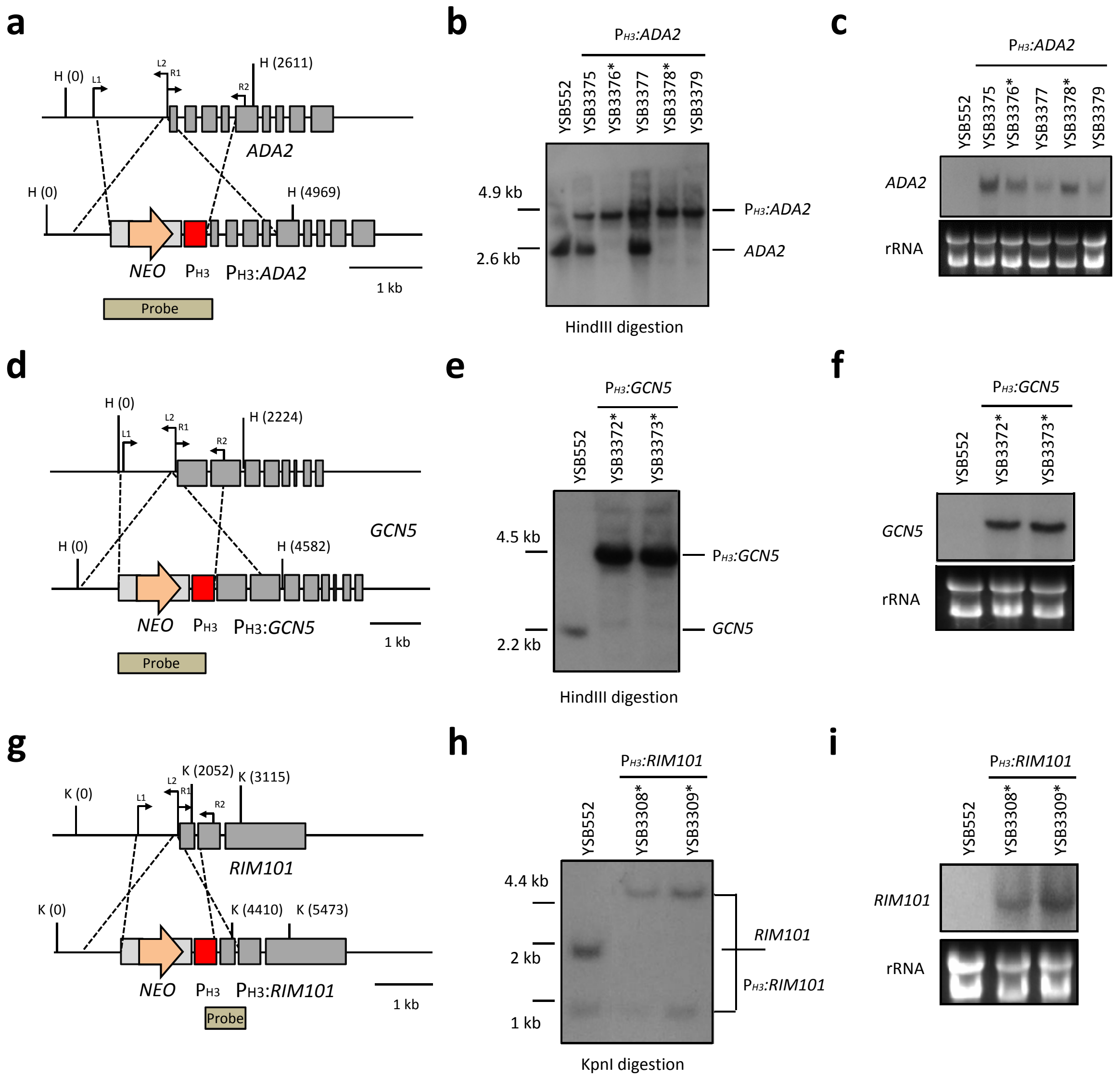

Supplementary Figure S8. Construction of the constitutively *ADA2-, GCN5-,* and *RIM101*-overexpressing strains from the *ire1*Δ mutant. (a, d, and g) A diagram of construction of the constitutively *ADA2-, GCN5-,* and *RIM101*-overexpressing strains using the H3 promoter. (b, e, and h) The correct genotype of the P*H3:ADA2,* P*H3:GCN5,* and P*H3:RIM101* strains in the strain (P*H3:ADA2*: YSB3376 and YSB3378, P*H3:GCN5*: YSB3372 and YSB3373, and P*H3:RIM101*:YSB3308 and YSB3309) was confirmed by Southern blot analysis using genomic DNAs digested with the indicated restriction enzyme. (c, f, and i) Northern blot analysis for measurement of *ADA2, GCN5,* and *RIM101* expression levels. Each membrane was hybridised with *ADA2-*, *GCN5-*, and *RIM101*-specific probes, washed, and developed. Data from ethidium bromide staining of rRNA was used for loading control. Strains marked with asterisk were used in this study.

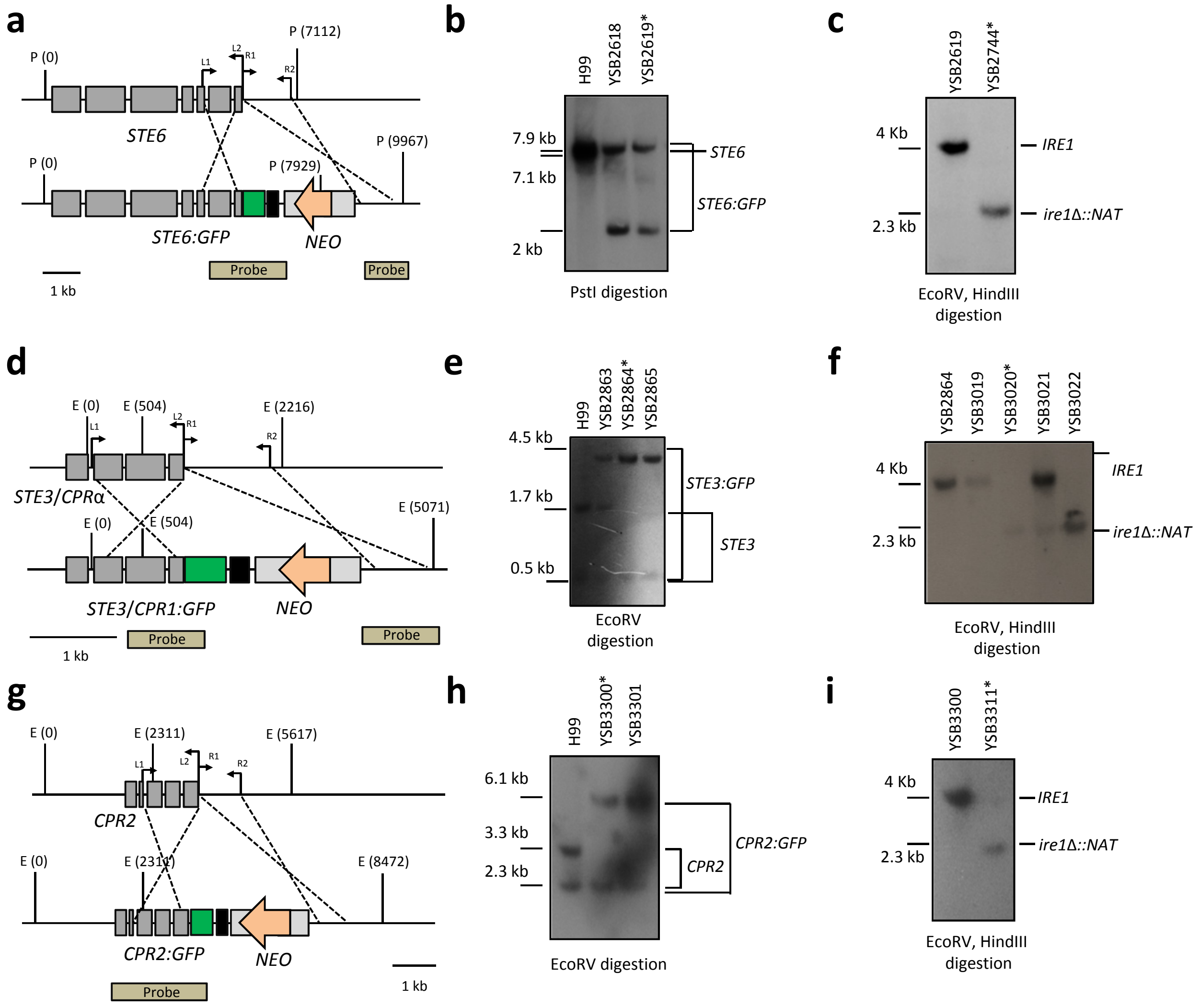

Supplementary Figure S9. Construction of the *STE6:GFP, STE3/CPRα:GFP*, and *CPR2:GFP* strains and *ire1Δ* mutant in each Gfp strain background. (a, d, and g) The scheme for construction of the *STE6:GFP*, *STE3/CPRα:GFP*, and *CPR2:GFP* strains. (b, e, and h) The correct genotype of the *STE6:GFP, STE3/CPRα:GFP*, and *CPR2:GFP* strains in the strain (*STE6:GFP*: YSB2618 and YSB2619, *STE3/CPRα:GFP*: YSB2864 and YSB2865, and *CPR2:GFP*: YSB3300 and YSB3301) was confirmed by Southern blot analysis using genomic DNAs digested with the indicated restriction enzyme. (c, f, and i) Southern blot analysis for verification of the *ire1Δ* mutant in each Gfp strain background. Strains marked with asterisk were used in this study.

Supplementary Table S1. Strains used in this study

Strain	Genotype	Parent	Reference
H99	*MAT*α		ref. 1
KN99**a**	*MAT***a**		ref. 2
JEC21	*MAT*α		ref. 3
JEC20	*MAT***a**		ref. 3
XL280	*MAT*α		ref. 4
YSB552	*MAT*α *ire1*Δ*::NAT-STM#224*	H99	ref. 5
YSB1000	*MAT*α *ire1*Δ*::NAT-STM#224 IRE1-NEO*	YSB552	ref. 5
YSB723	*MAT*α *hxl1*Δ*::NAT-STM#295*	H99	ref. 5
YSB762	*MAT*α *hxl1*Δ*::NAT-STM#295 HXL1-NEO*	YSB723	ref. 5
YSB737	*MAT*α *HXL1*^{us}*-NEO*	H99	ref. 5
YSB742	*MAT*α *HXL1*^{s}*-NEO*	H99	ref. 5
YSB1126	*MAT*α *ire1*Δ*::NAT-STM#224 HXL1*^{us}*-NEO*	YSB552	ref. 5
YSB1127	*MAT*α *ire1*Δ*::NAT-STM#224 HXL1*^{s}*-NEO*	YSB552	ref. 5
YSB1225	*MAT*α *hxl1*Δ*::NAT-STM#295 HXL1*^{s}*-NEO*	YSB723	ref. 5
YSB119	*MAT*α *aca1*Δ*::NAT-STM#43 ura5 ACA1-URA5*	YSB108	ref. 6
YSB121	*MAT*α *aca1*Δ*::NEO ura5 ACA1-URA5*	YSB109	ref. 6
H99 crg1	*MAT*α *ura5 crg1*Δ*::URA5*	F99	ref. 7
PPW196	*MAT***a** *ura5 crg1*Δ*::URA5*	F99**a**	ref. 7
YSB53	*MATa ras1*Δ*::NAT-STM#150*	H99	ref. 6
YSB127	*MATa cpk1*Δ*::NAT-STM#184*	H99	ref. 8
YSB1366	*MATa rim101*Δ*::NAT-STM#208*	H99	ref. 9
YSB550	*MAT***a** *ire1*Δ*::NEO*	KN99**a**	This study
YSB850	*MAT***a** *hxl1*Δ*::NEO*	KN99**a**	This study
KW308	*MAT*α *ire1*Δ*::NAT-STM#224 hxl1*Δ*::NEO*	YSB552	This study
KW309	*MAT*α *ire1*Δ*::NAT-STM#224 hxl1*Δ*::NEO*	YSB552	This study
KW350	*MAT***a** *ire1*Δ*::NEO hxl1*Δ*::NAT-STM#56*	YSB550	This study
KW351	*MAT***a** *ire1*Δ*::NEO hxl1*Δ*::NAT-STM#56*	YSB550	This study
YSB1741	*MAT*α P$_{H3}$*:KAR2-NEO ire1*Δ*::NAT-STM#224*	YSB552	ref. 10
YSB3194	*MAT***a** P$_{H3}$*:KAR2-NAT ire1*Δ*::NEO*	YSB550	This study
YSB3308	*MAT*α P$_{H3}$*:RIM101-NEO ire1*Δ*::NAT-STM#224*	YSB552	This study
YSB3309	*MAT*α P$_{H3}$*:RIM101-NEO ire1*Δ*::NAT-STM#224*	YSB552	This study
YSB3372	*MAT*α P$_{H3}$*:GCN5-NEO ire1*Δ*::NAT-STM#224*	YSB552	This study
YSB3373	*MAT*α P$_{H3}$*:GCN5-NEO ire1*Δ*::NAT-STM#224*	YSB552	This study
YSB3376	*MAT*α P$_{H3}$*:ADA2-NEO ire1*Δ*::NAT-STM#224*	YSB552	This study
YSB3378	*MAT*α P$_{H3}$*:ADA2-NEO ire1*Δ*::NAT-STM#224*	YSB552	This study
YSB1008	*MAT*α *ura5 crg1*Δ*::URA5 ire1*Δ*::NAT-STM#125*	H99 crg1	This study
YSB1009	*MAT*α *ura5 crg1*Δ*::URA5 ire1*Δ*::NAT-STM#125*	H99 crg1	This study
YSB1010	*MAT***a** *ura5 crg1*Δ*::URA5 ire1*Δ*::NAT-STM#125*	PPW196	This study
YSB1011	*MAT***a** *ura5 crg1*Δ*::URA5 ire1*Δ*::NAT-STM#125*	PPW196	This study
YSB2505	*MAT*α *ire1*Δ*::NAT-STM#224 cpk1*Δ*::NEO*	YSB552	This study
YSB2507	*MAT*α *ire1*Δ*::NAT-STM#224 ras1*Δ*::NEO*	YSB552	This study
YSB2619	*MAT*α *STE6:GFP-NEO*	H99	This study
YSB2744	*MAT*α *STE6:GFP-NEO ire1*Δ*::NAT-STM#169*	YSB2619	This study
YSB2864	*MAT*α *STE3:GFP-NEO*	H99	This study
YSB3020	*MAT*α *STE3:GFP-NEO ire1*Δ*::NAT-STM#169*	YSB2864	This study
YSB3000	*MAT*α *CPR2:GFP-NEO*	H99	This study
YSB3311	*MAT*α *CPR2:GFP-NEO ire1*Δ*::NAT-STM#169*	YSB3000	This study
YSB2886	*MAT*α *ire1*Δ*::NAT-STM#273*	JEC21	This study
YSB2026	*MAT***a** *ire1*Δ*::NEO*	JEC20	This study
YSB2030	*MAT*α *hxl1*Δ*::NAT-STM#58*	JEC21	This study
YSB1985	*MAT***a** *hxl1*Δ*::NEO*	JEC20	This study
YSB2144	*MAT*α *ire1*Δ*::NAT-STM#273*	XL280	This study
YSB2145	*MAT*α *ire1*Δ*::NAT-STM#273*	XL280	This study
YSB2147	*MAT*α *hxl1*Δ*::NAT-STM#43*	XL280	This study
YSB2148	*MAT*α *hxl1*Δ*::NAT-STM#43*	XL280	This study

KW345	*MATα ire1Δ::NAT-STM#273 hxl1Δ::NEO*	YSB2145	This study
KW346	*MATα ire1Δ::NAT-STM#273 hxl1Δ::NEO*	YSB2145	This study
YSB3596	*MATα ire1Δ::NAT-STM#273 IRE1-NEO*	YSB2144	This study
YSB3492	*MATα* P_{H3}:*KAR2-NEO*	XL280	This study
YSB3493	*MATα* P_{H3}:*KAR2-NEO*	XL280	This study
YSB3499	*MATα* P_{H3}:*KAR2-NEO ire1Δ::NAT-STM#273*	YSB2145	This study
YSB3500	*MATα* P_{H3}:*KAR2-NEO ire1Δ::NAT-STM#273*	YSB2145	This study

Each *NAT-STM#* indicates the Nat^{r} marker with a unique signature tag.

Supplementary Table S2. Primers used in this study

Primer Name	Sequence (5'—3')	Comment
B79	TGTGGATGCTGGCGGAGGATA	Screening primer on ACT promoter
B1026	GTAAAACGACGGCCAGTGAGC	M13 forward (extended)
B1027	CAGGAAACAGCTATGACCATG	M13 reverse (extended)
B1454	AAGGTGTTCCCCGACGACGAATCG	NSL
B1455	AACTCCGTCGCGAGCCCCATCAAC	NSR
B1886	TGGAAGAGATGGATGTGC	NSL-NEO
B1887	ATTGTCTGTTGTGCCCAG	NSR-NEO
B4017	GCATGCAGGATTCGAGTG	H3 promoter- left flanking primer 1
B4018	GTGATAGATGTGTTGTGGTG	H3 promoter- right flanking primer 2
B354	GCATGCAGGATTCGAGTG	GFPht-NEO – left flanking primer 1
B5665	GGTGGCGGTGGCTCTGTGAGC	GFPht-NEO – left flanking primer 2
B1648	AGGAATACGAGGTTTATCGG	*IRE1* - screening primer (for serotype A)
B1644	GCCCCATCATCATAATCAC	*IRE1* - left flanking primer 1 (for serotype A)
B1645	GCTCACTGGCCGTCGTTTTACACTATGTGTCCATCTGAGGC	*IRE1* - left flanking primer 2 (for serotype A)
B1646	CATGGTCATAGCTGTTTCCTGAGTGAGTTGAGGGAGGAAAG	*IRE1* - right flanking primer 1 (for serotype A)
B1647	GAAGAAGAGCGTCAAGAAGG	*IRE1* - right flanking primer 2 (for serotype A)
B1683	AGCATTAGGGGTGTAGGTG	*IRE1* – Southern blot probe primer 1 (for serotype A)
B1880	AACTCTTCTCAGCCTTCGG	*HXL1* - screening primer (for serotype A)
B1881	GTTTGAGGCTGGTAAAAAGG	*HXL1* - left flanking primer 1 (for serotype A)
B1882	GCTCACTGGCCGTCGTTTTACATGGGGAATGAAAGCGTG	*HXL1* - left flanking primer 2 (for serotype A)
B1883	CATGGTCATAGCTGTTTCCTGAAGGGGCGAGAGTAGTTCAG	*HXL1* - right flanking primer 1 (for serotype A)
B1884	GACTGTAAAGGAGGGCATAAG	*HXL1* - right flanking primer 2 (for serotype A)
B1885	CGTTCTCCGTCTTGATAGC	*HXL1* – Southern blot probe primer 1 (for serotype A)
B2167	CAAGGTTGACGAGATGAGTATG	*IRE1* - screening primer (for serotype D and XL280)
B2168	CTTTCTTTTTCCGCCTACC	*IRE1* - left flanking primer 1 (for serotype D and XL280)
B2169	TCACTGGCCGTCGTTTTAC GTCGCCGAGAGAATAAAATC	*IRE1* - left flanking primer 2 (for serotype D and XL280)
B2170	CATGGTCATAGCTGTTTCCTG TGAGTTGAGGGAGGAAAGTC	*IRE1* - right flanking primer 1 (for serotype D and XL280)
B2171	AAGGACACTATCCGTTCCG	*IRE1* - right flanking primer 2 (for serotype D and XL280)
B4948	GATTTGGGTCGGAGATTC	*IRE1* – Southern blot probe primer 1 (for serotype D and XL280)
B4769	GGAGTGAAAGCAGGAGTTG	*HXL1* - screening primer (for serotype D and XL280)
B4770	CCATCGTTCGGTATGCTAC	*HXL1* - left flanking primer 1 (for serotype D and XL280)
B4771	TCACTGGCCGTCGTTTTACAGTGGAAATAGGTGCGATG	*HXL1* - left flanking primer 2 (for serotype D and XL280)
B4772	CATGGTCATAGCTGTTTCCTGTTGGAAGGGAGGAAATGC	*HXL1* - right flanking primer 1 (for serotype D and XL280)
B4773	GGGTTAGTAGGAAGAAGTAGGC	*HXL1* - right flanking primer 2 (for serotype D and XL280)
B4774	CGTTGGCGAAGGACAATAC	*HXL1* – Southern blot probe primer 1 (for serotype D and XL280)
B3550	TCCCAATCTACTGACCTATCG	*KAR2* – 5' screening primer (H3 promoter replacement for serotype A)
B3551	CGTAGGGTATGTCTCTGATGAG	*KAR2* – left flanking primer 1 (H3 promoter replacement for serotype A)
B4270	CACTCGAATCCTGCATGCGGTGGCAAAAGTCTTGAGGA	*KAR2* – left flanking primer 2 (H3 promoter replacement for serotype A)
B4264	CAAGACCTCAAAGACACCG	*KAR2* – right flanking primer 1 (H3 promoter replacement for serotype A)
B4271	ACCACAACACATCTATCACATGGCATACCCTTCAAGAAT	*KAR2* – right flanking primer 2 (H3 promoter replacement for serotype A)
B3555	CAAGCAGGGACAGTAACAAC	*KAR2* – Southern blot probe primer 1 (for serotype A)
B4978	AGGCAGTCTGGAGTGTCATC	*KAR2* – Northern blot probe primer 1 (for serotype A)
B2182	TCAGCAGCAGCAGGTAAAC	*ADA2* – 5' screening primer (H3 promoter replacement for serotype A)
B2183	GGATGATGGAATCGTATGC	*ADA2* – left flanking primer 1 (H3 promoter replacement for serotype A)
B6399	CACTCGAATCCTGCATGCACTCGTTTGTTGATGCCTTT	*ADA2* – left flanking primer 2 (H3 promoter replacement for serotype A)
B6400	ACCACAACACATCTATCACATGACTGTCACGCAGAGGAA	*ADA2*– right flanking primer 1 (H3 promoter replacement for serotype A)

B2187	TTCATCTGGAGGACGAGTG	*ADA2* – right flanking primer 2 (H3 promoter replacement for serotype A)
B4269	CCCAGACCGTTTTGAATG	*ADA2*– Southern blot probe primer 1 (for serotype A)
B2183	GGATGATGGAATCGTATGC	*ADA2*– Southern blot probe primer 2 (for serotype A)
B2187	TTCATCTGGAGGACGAGTG	*ADA2* – Northern blot probe primer 1 (for serotype A)
B6400	ACCACAACACATCTATCACATGACTGTCACGCAGAGGAA	*ADA2* – Northern blot probe primer 2 (for serotype A)
B2986	CACGGCAACTTATGCTCTC	*RIM101* – 5' screening primer (H3 promoter replacement)
B2982	CATCAGTCTTGCTTCTTCTGC	*RIM101* – left flanking primer 1 (H3 promoter replacement)
B6201	CACTCGAATCCTGCATGCCTTGGCCTTGCTGTTAACTT	*RIM101* – left flanking primer 2 (H3 promoter replacement)
B6202	ACCACAACACATCTATCACATGGCTTACCCAATTCTCCC	*RIM101*– right flanking primer 1 (H3 promoter replacement)
B6203	AGCACCAAAAGGTTCAGC	*RIM101* – right flanking primer 2 (H3 promoter replacement)
B4980	ACTCGGTGTTGGTGAAACGG	*RIM101*– Southern blot probe primer 1
B4980	ACTCGGTGTTGGTGAAACGG	*RIM101* – Northern blot probe primer 1
B6170	GCCAAATCTTCTCAGCACTC	*RIM101* – Northern blot probe primer 2
B6401	CAAAAATGGCGTCAGGTC	*GCN5* – 5' screening primer (H3 promoter replacement)
B6402	CAATGATGAATGACCACGAC	*GCN5* – left flanking primer 1 (H3 promoter replacement)
B6403	CACTCGAATCCTGCATGCAGCAGAAACGGAAAGGCTTA	*GCN5* – left flanking primer 2 (H3 promoter replacement)
B6404	ACCACAACACATCTATCACATGGCGCCAAAACCACGTCGT	*GCN5*– right flanking primer 1 (H3 promoter replacement)
B6405	TCAGAGTGGGTAGATTCGC	*GCN5* – right flanking primer 2 (H3 promoter replacement)
B6406	CGTCTTCGTCTTCATTGC	*GCN5* – Southern blot probe primer 1
B6402	CAATGATGAATGACCACGAC	*GCN5* – Southern blot probe primer 2
B6405	TCAGAGTGGGTAGATTCGC	*GCN5* – Northern blot probe primer 1
B6406	CGTCTTCGTCTTCATTGC	*GCN5* – Northern blot probe primer 2
B6632	TGGCGGAGAAACAGTAGAG	*KAR2* – 5' screening primer (H3 promoter replacement for XL280)
B6633	GGCAACCCTCATCTACTGAC	*KAR2* – left flanking primer 1 (H3 promoter replacement for XL280)
B6634	CACTCGAATCCTGCATGCCCCGGGCCTTTTTACCCTTT	*KAR2* – left flanking primer 2 (H3 promoter replacement for XL280)
B6635	ACCACAACACATCTATCACATGAAATGGCGAGTGAGGCG	*KAR2* – right flanking primer 1 (H3 promoter replacement for XL280)
B6644	CACTATTACCCACAAGCGG	*KAR2* – right flanking primer 2 (H3 promoter replacement for XL280)
B6637	AAGTCGTTCCTCCTCAGTG	*KAR2* – Southern blot probe primer 1 (for XL280)
B5661	CAGTCTAACTTTTGGCAGTGG	*STE6*:*GFP* tagging left flanking primer 1
B5662	GCTCACAGAGCCACCGCCACCCTCCCATTCTCCCGTCTTCATCA	*STR6*:*GFP* tagging left flanking primer 2
B5663	GCCACTCGAATCCTGCATGCATTATAGAGGTTGTCTTGC	*STE6*:*GFP* tagging right flanking primer 1
B5664	GCTCGGGTAAGATACTGAGG	*STE6*:*GFP* tagging right flanking primer 2
B5660	GAGGGTTGCCTTTGTTTG	*STE6*:*GFP* tagging screening primer
B5741	TTGGTTTCATAGCCCTGC	*STE6*:*GFP* tagging Southern blot probe primer
B5897	TTTTCATCGGAGTCCCTG	*STE3*/*CPRα*:*GFP* tagging left flanking primer 1
B5898	GCTCACAGAGCCACCGCCACCAACGACAGCACGCTCGACCGA	*STE3*/*CPRα*:*GFP* tagging left flanking primer 2
B5899	GCCACTCGAATCCTGCATGCTTCCACGATGGTTGATGTAAA	*STE3*/*CPRα*:*GFP* tagging right flanking primer 1
B5900	GACAAGAATGCGATGTGG	*STE3*/*CPRα*:*GFP* tagging right flanking primer 2
B4790	CTTCTACCTCTGCCTCTTCAC	*STE3*/*CPRα*:*GFP* tagging screening primer
B5959	CGTCTCCCATCTCACTTTG	*STE3*/*CPRα*:*GFP* tagging Southern blot probe primer
B5943	TATGATTCTTCCGCTCGC	*CPR2:GFP* tagging left flanking primer 1
B5902	GCTCACAGAGCCACCGCCACCAACCATTGATGAGCTAGTCTT	*CPR2:GFP* tagging left flanking primer 2
B5903	GCCACTCGAATCCTGCATGCAATGTCCATTTGTTGGGACCG	*CPR2:GFP* tagging right flanking primer 1
B5904	GACCAAGCAAGAAGAAGATG	*CPR2:GFP* tagging right flanking primer 2
B4792	CCATCTGTCATCGCTTTG	*CPR2:GFP* tagging diagnostic primer
B4793	CAGTGGTAGCAGAGGAATAGG	*CPR2:GFP* tagging Southern blot probe primer
B1894	TTTTACGCTTTTTGCAGATTCCGCCAAA	*MFα1* primer 1 for Northern blot
B1895	GACCACTGTTTCTTTCGTTCT	*MFα1* primer 2 for Northern blot
J94	CGCCTTCACTGCCATCTTC	*MFα1* primer 1 for qRT analysis
J95	ACAAAGGGTCATGCCACCGG	*MFα1* primer 2 for qRT analysis
B679	CGCCCTTGCTCCTTCTTCTATG	*ACT1* primer 1 for qRT analysis
B680	GACTCGTCGTATTCGCTCTTCG	*ACT1* primer 2 for qRT analysis

JOHE12039	CTGTAGAAGATGTGAGTTTGGG	*CPK1* - left flanking primer 1
JOHE12040	CTGGCCGTCGTTTTACTGATTGATGAGAGATACGGG	*CPK1* - left flanking primer 2
JOHE12041	GTCATAGCTGTTTCCTGGGCGGAGAAATAGAGGTTG	*CPK1* - right flanking primer 1
JOHE12042	CGCACAAGAAGTAAGAGGTG	*CPK1* - right flanking primer 2
JOHE12043	GGCTATGGACCGTATTCAC	*CPK1* – screening primer
JOHE12045	TATCTCACAAGCCACTCCC	*CPK1* – Southern blot probe primer 1
JOHE12046	ATGCTGCTCACCGTTAGTC	*CPK1* – Southern blot probe primer 2
JOHE11600	AAGACTGGTTCAGCAGAGC	*RAS1* - left flanking primer 1
JOHE11601	CTGGCCGTCGTTTTACCTAAATGGGGATGGTTCG	*RAS1* - left flanking primer 2
JOHE11602	GTCATAGCTGTTTCCTGAAACATCCGCCAAGCAAC	*RAS1* - right flanking primer 1
JOHE11603	GAGCAAAATGAGGAACTTGG	*RAS1* - right flanking primer 2
JOHE11604	TGTGCTTTACCAGGCAGTCG	*RAS1* – screening primer
JOHE11606	CACACTCTTTGTCCTTCCG	*RAS1* – Southern blot probe primer 1
JOHE11607	ACCGTATTCTTCTTG ACC G	*RAS1* – Southern blot probe primer 2

References

1. Perfect, J. R., Ketabchi, N., Cox, G. M., Ingram, C. W. & Beiser, C. L. Karyotyping of *Cryptococcus neoformans* as an epidemiological tool. *J. Clin. Microbiol.* **31**, 3305-3309 (1993).

2. Nielsen, K. *et al.* Sexual cycle of *Cryptococcus neoformans* var. *grubii* and virulence of congenic **a** and a isolates. *Infect. Immun.* **71**, 4831-4841 (2003).

3. Kwon-Chung, K. J., Edman, J. C. & Wickes, B. L. Genetic association of mating types and virulence in *Cryptococcus neoformans*. *Infect. Immun.* **60**, 602-605 (1992).

4. Lin, X., Hull, C. M. & Heitman, J. Sexual reproduction between partners of the same mating type in *Cryptococcus neoformans*. *Nature* **434**, 1017-1021 (2005).

5. Cheon, S. A. *et al.* Unique evolution of the UPR pathway with a novel bZIP transcription factor, Hxl1, for controlling pathogenicity of *Cryptococcus neoformans*. *PLoS Pathog.* **7**, e1002177 (2011).

6. Bahn, Y. S., Hicks, J. K., Giles, S. S., Cox, G. M. & Heitman, J. Adenylyl cyclase-associated protein Aca1 regulates virulence and differentiation of *Cryptococcus neoformans* via the cyclic AMP-protein kinase A cascade. *Eukaryot. Cell* **3**, 1476-1491 (2004).

7. Wang, P., Cutler, J., King, J. & Palmer, D. Mutation of the regulator of G protein signaling Crg1 increases virulence in *Cryptococcus neoformans*. *Eukaryot. Cell* **3**, 1028-1035 (2004).

8. Bahn, Y. S., Geunes-Boyer, S. & Heitman, J. Ssk2 mitogen-activated protein kinase kinase kinase governs divergent patterns of the stress-activated Hog1 signaling pathway in *Cryptococcus neoformans*. *Eukaryot. Cell* **6**, 2278-2289 (2007).

9. Jung, K. W. *et al.* Systematic functional profiling of transcription factor networks in *Cryptococcus neoformans*. *Nat. Commun.* **6**, 6757 (2015)

10. Jung, K. W., Kang, H. A. & Bahn, Y. S. Essential roles of the Kar2/BiP molecular chaperone downstream of the UPR pathway in *Cryptococcus neoformans*. *PLoS ONE* **8**, e58956 (2013).
